# Active control of viscous fingering using electric fields

**DOI:** 10.1038/s41467-019-11939-7

**Published:** 2019-09-05

**Authors:** Tao Gao, Mohammad Mirzadeh, Peng Bai, Kameron M. Conforti, Martin Z. Bazant

**Affiliations:** 1 Department of Chemical Engineering, Massachusetts Institute of Technology, Cambridge, MA 02139 USA; 2 Department of Mathematics, Massachusetts Institute of Technology, Cambridge, MA 02139 USA; 3 Present Address: Department of Energy, Environmental, and Chemical Engineering, Washington University in St. Louis, St. Louis, MO 63130 USA

**Keywords:** Applied mathematics, Fluid dynamics

## Abstract

Viscous fingering is a widely observed phenomenon, in which finger-like instabilities occur at the interface of two fluids, whenever a less viscous phase displaces a more viscous phase. This instability is notoriously difficult to control, especially for given viscosity ratio and geometry. Here we demonstrate experimentally the active control of viscous fingering of two given liquids, for given geometry and flow rate in a Hele-Shaw cell. The control is realized by taking advantage of electro-osmotic flows along the surfaces confining the fluid, via applying an external electric field. Depending on the direction of electric field, the induced secondary electro-osmotic flows either assist or oppose the hydraulic flow, effectively reducing or increasing the flow resistance, leading to the control of interface stability. The mechanism of apparent “electrokinetic thinning/thickening” is proposed to explain the experimental observations. Theoretical predictions of linear stability are confirmed experimentally for a broad range of immiscible electrolyte displacements.

## Abstract (web summary)

When a viscous fluid is displaced by a less viscous phase between two plates, a finger-like instability occurs. Here the authors demonstrate how applying an external electric field can suppress the fingering instability without the need to alter the fluid flow rate or the gap geometry.

## Introduction

Interfacial instabilities are prevalent in nature and often lead to captivating patterns^1^. Snowflakes are familiar examples, which form when the rate of ice formation is limited by heat diffusion^2,3^. Similar “dendritic” patterns commonly arise in solidification^4–7^, as well as electrodeposition of metals, such as copper^8^ or lithium^9^, limited by electro-diffusion. In biology, diverse patterns of bacterial colonies^10,11^ or fractal shapes of retinal vessels^12^ have been attributed to mass transfer limitations of nutrition or oxygen.

Interfacial instabilities are usually undesirable in practical applications. For example, dendritic growth is a major safety issue for rechargeable batteries^13^, while “viscous fingering” reduces the efficiency of enhanced oil recovery by water flooding^14^. Nevertheless, there are situations where interfacial instabilities may be beneficial, e.g. in enhancing CO_2_ mixing in saline aquifers for carbon sequestration^15^, increasing mixing efficiency in microfluidics^16^, or patterning soft materials^17^. Therefore, the notion of “active control”, i.e. to deliberately suppress or enhance interfacial instabilities, is quite attractive.

Viscous fingering is perhaps the most fundamental—and difficult to control—interfacial instability. Finger-like patterns form when a lower viscosity fluid (e.g. water) displaces a more viscous one (e.g. oil), effectively creating paths of lower hydraulic resistance^18^. This problem was originally studied by Hill^19^ in the early 1950s, and soon followed by Saffman and Taylor^20^ and Chuoke et al.^21^. In particular, Saffman and Taylor first conducted experiments in a “Hele-Shaw cell”, a flow apparatus consisting of two parallel glass plates separated by a thin gap, and performed linear stability analysis to show that the onset of instability is controlled by a single parameter, the viscosity ratio. This leaves very little room to control stability, once the fluids and geometry are specified.

In recent years, several strategies have been pursued to manipulate the conditions for viscous fingering. Examples of passive control have exploited geometrical heterogeneity^22,23^, elastic substrates^24–26^, and modified wettability^27,28^. Active control of viscous fingering has also been achieved by adjusting the flow rate^29^ or gap thickness^30,31^ over time during the experiment. In many applications, however, it would preferable to somehow control the instability for a given flow rate and geometry.

Here, we demonstrate that active control of viscous fingering in a Hele-Shaw cell is possible by applying external electric fields parallel to the flow direction. We also introduce the concept of apparent “electrokinetic thickening/thinning” to explain the observed phenomena. When electric fields are present, the pressure must adjust to compensate for the opposing/assisting electro-osmotic flow, which leads to increased/reduced flow resistance, as if fluid viscosities were effectively increased/decreased. The interface stability is therefore determined by the strength of apparent electrokinetic thickening/thinning effect in both fluids. In our experiment, the defending water phase has much larger permittivity and surface charge than the invading oil phase, which leads to stronger electro-osmotic flow (and therefore stronger apparent electrokinetic thickening/thinning effect) in the water phase. As a result, positive currents help to stabilize the interface motion while negative currents destabilize it. The extent of this active control depends on the magnitude of the applied electric field.

## Results

### Theory

**Fig. 1:**
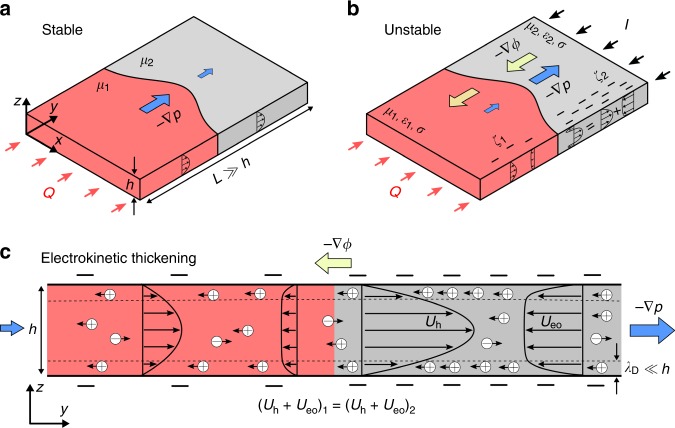
Electric fields can alter the interfacial stability in a Hele-Shaw cell. **a** Hydrodynamically Stable: an interface moves in response to a pressure gradient and follows the path of least resistance. When the invading fluid has a higher viscosity (*μ*_1_ > *μ*_2_), the hydraulic resistance is stronger behind the interface and the motion is stable. **b** Becomes Unstable with External Electric Field: Applying an electric field drives an additional “electro-osmotic” flow. When the electro-osmotic flow is in the opposite direction of hydraulic flow, the overall resistance is enhanced due to apparent “electrokinetic thickening”. If electro-osmotic flows are stronger ahead of the interface, electrokinetic thickening destabilizes the interface motion for sufficiently large electric fields. **c** Mechanism: apparent electrokinetic thickening: Glass surfaces are negatively charged in aqueous solutions due to dissociation of silanol groups. An applied electric field acts on the positive ions in the EDL (thickness *λ*_*D*_ ≪ *h*) and drives an electro-osmotic flow (*U*_eo_) in the opposite direction as hydraulic flow (*U*_h_). To maintain the same total flow rate in each phase, pressure gradients must adjust to compensate for the electro-osmotic flows, causing an apparent enhancement of the viscosity which we refer to as apparent “electrokinetic thickening”. Conversely, apparent “electrokinetic thinning” is caused if the electro-osmotic flows are in the same direction as hydraulic flows

Consider the interface between two immiscible liquids, as it moves in the small gap between two parallel plates (Fig. 1). Intuitively, the interface should deform, if necessary, to follow the path of least resistance. If the invading fluid has a lower viscosity (*μ*_1_ < *μ*_2_), a small perturbation gradually grows since it locally reduces the hydraulic resistance and introduces a preferred direction for the interface motion. Conversely, when the invading liquid has a higher viscosity (*μ*_1_ > *μ*_2_), any perturbation decays as it locally increases the hydraulic resistance and inhibits further motion. This simple physical argument captures the essence of viscous fingering and is consistent with detailed linear stability analysis^18^. Therefore, in the absence of electric fields, interfacial stability depends only upon a single parameter, the viscosity ratio:

1 $${\mathrm{Stable}}:M = \frac{{\mu _1}}{{\mu _2}} > 1$$

but the situation changes if the interface is driven by multiple forces.

Electric forces come to mind, since most surfaces become charged in contact with liquids, often due to dissociation of ionic surface groups. For instance, glass surfaces are negatively charged at high pH due to deprotonation of silanol groups (SiOH ↔ SiO^−^ + H ^+^). When ions are present in the solution, the surface charge is screened by a diffuse cloud of excess counter-ions that forms the electrical double layer (EDL). Due to the presence EDL, the applied electric field drives electrokinetic phenomena of coupled ion transport and fluid flow^32^. In particular, an electric field parallel to the surface exerts a net force on ions in the EDL and drives a secondary electro-osmotic flow, in addition to the pressure-driven flow (see Fig. 1). If the electro-osmotic flow is in the same direction as the pressure-driven flow, the effective hydraulic resistance is reduced, while oppositely directed flow increase the effective resistance. Therefore, it should be possible to manipulate interfacial stability confined between charged surfaces by controlling the electro-osmotic flow velocity with an external electric field.

Indeed, a simple analysis of this mechanism leads to the generalized stability condition^33^, as follows. In a Hele-Shaw cell, the depth-averaged hydraulic velocity is related to the pressure gradient, **G** = −∇*p*, via **u**_h_ = *K*_h_**G**, where *K*_h_ = *h*^2^/12*μ* is the hydraulic conductivity and *h* is the gap thickness. Similarly, the electro-osmotic velocity is related to the electric field, **E** = −∇*ϕ*, via **u**_eo_ = *K*_eo_**E**, where *K*_eo_ is the electro-osmotic mobility. For thin EDLs (Debye length *λ*_*D*_ ~ 10 nm much smaller than gap thickness *h* ~ 200 μm), the electro-osmotic mobility is given by the Helmholtz-Smoluchowski relation *K*_eo_ = −*εζ*/*μ*^32^. Here, *ε* and *ζ* are the permittivity coefficient and the surface potential, respectively. The total depth-averaged velocity is then given by **u** = **u**_h_ + **u**_eo_. Since the overall flow resistance is directly proportional to the pressure gradient, stable displacement is possible if *G*_1_ > *G*_2_, which yields:

2 $${\mathrm{Stable}}:U(\mu _1 - \mu _2) + E\left( {\varepsilon _1\zeta _1 - \varepsilon _2\zeta _2} \right) > 0.$$

Therefore, it is clear electric fields affect the interfacial stability. When the electro-osmotic flow is in the opposite direction of the hydraulic flow (*U*_eo_/*U* < 0), the pressure gradients must adjust to compensate for the electro-osmotic flow which manifests itself as an extra resistance (see Fig. 1). We refer to this phenomenon as apparent “electrokinetic thickening”, as if the viscosities of the fluids are increased. Conversely, by apparent “electrokinetic thinning” we refer to the reduction in the overall resistance when electro-osmotic and hydraulic flows are in the same directions. The degree of apparent thickening or thinning depends on the strength of electro-osmotic flow. We emphasize that electrokinetic effects do not change the molecular viscosity of the solvent and that the thinning or thickening effects are merely “apparent”.

In our experiments, we use oil as the invading fluid and water-glycerol mixture as the defending fluid, in which the water phase (fluid 2) is much more polar and has a more negative surface potential than the oil phase (fluid 1) (*ε*_2_*ζ*_2_ < *ε*_1_*ζ*_1_ < 0). Negative electric field (*E* < 0) leads to apparent electrokinetic thickening (*U*_eo_/*U* < 0), and the interface is destabilized because the stronger electro-osmotic flows in the defending water phase makes it more resistant. In contrast, positive electric field (*E* > 0) leads to apparent electrokinetic thinning (*U*_eo_/*U* > 0), and the interface is stabilized because the water phase becomes even less resistant. Since we conduct our experiments under constant flow rate (*Q*) and electric current (*I*), it is convenient to express the stability criteria in terms of a non-dimensional current $\tilde I$:

3 $${\mathrm{Stable}}:\tilde I = \frac{I}{Q}\frac{{\varepsilon _2\zeta _2 - \varepsilon _1\zeta _1}}{{\sigma (\mu _2 + \mu _1)}} < \frac{{M - 1}}{{M + 1}} = \tilde I_{{\mathrm{cr}}},$$

where *σ* is the electrical conductivity of both liquids. Equation (3) states that for any viscosity ratio, there is a critical current beyond which the stability is dominated by the electro-osmotic flow. The overall stability could thus be understood to depend on the relative strength of electro-osmotic flow, *U*_eo_/*U*, which is directly proportional to the electric current and is captured by the control parameter, $\tilde I \propto U_{{\mathrm{eo}}}/U$ (see Supplementary Notes 1, 2).

### Representative experiments

**Fig. 2:**
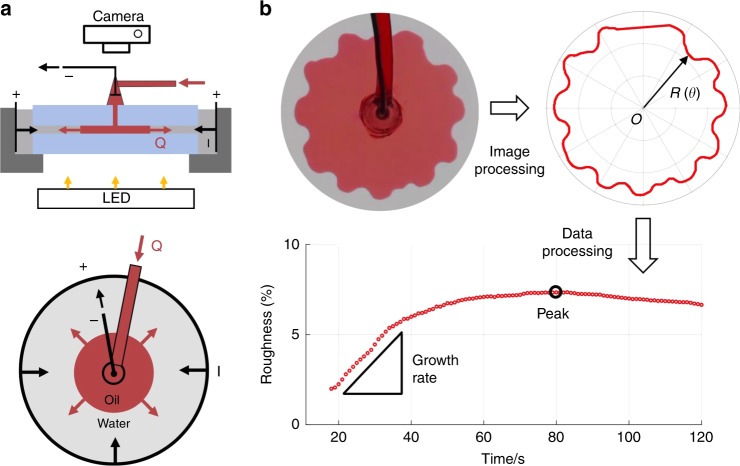
Experiment apparatus and data processing. **a** Experiment Apparatus: Top: side view. Bottom: top view. Electric current is applied through two electrodes, one in the center and another encircling the cell. Flow is always injected from center. Positive current is defined to be in the same direction as the flow (negative current shown in the schematic). **b** Image and data processing: the obtained pattern is digitized into a polar plot, and interfacial roughness is calculated for quantifying interface instability

Guided by Eq. (3), we design experiments to investigate the electrokinetic control of interfacial stability. A radial Hele-Shaw cell is constructed with copper electrodes installed both at the center and the circular outer edge of the cell (Fig. 2a). A digital camera is used to capture images from above, and the results are analyzed in MATLAB to compute interface roughness (Fig. 2b). The growth rate of roughness and peak roughness are used as the quantitative measure of instability (see Methods for the definition of roughness).

**Fig. 3:**
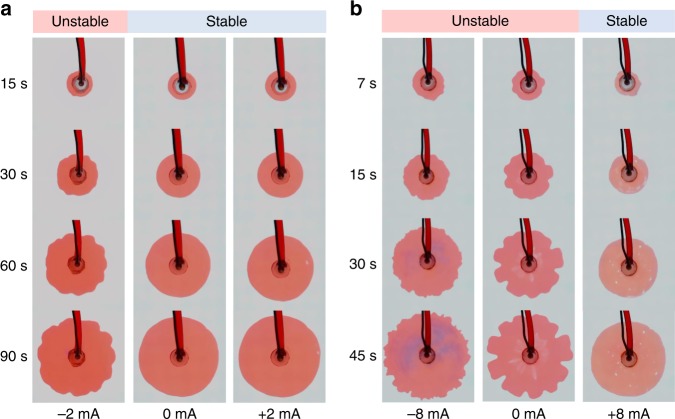
Representative examples of electrokinetic destabilization and stabilization. **a** Destabilization of a hydrodynamically stable displacement (*M* = *μ*_1_/*μ*_2_ = 1.98 > 1). *μ*_1_: viscosity of the inner phase (invading). *μ*_2_: viscosity of the outer phase (defending). **b** Stabilization of a hydrodynamically unstable displacement (*M* = 0.067 < 1). The videos of the evolution of the interfaces are provided in Supplementary Movie 1

For a hydrodynamically stable displacement, *M* > 1, i.e. moving into the low viscosity phase, the interface is inherently stable (Fig. 3a, *I* = 0 mA), but subject to control by applying an electric field. Specifically for oil pushing water, an electric field pointing toward water (*I* > 0) is stabilizing (apparent electrokinetic thinning), while an electric field pointing toward oil (*I* < 0) is destabilizing (apparent electrokinetic thickening). At a sufficiently large negative current, the destabilizing electro-osmotic flow fully counter balances the stabilizing viscous effects and the interface motion transitions from stable to unstable (Fig. 3a, *I* = −2 mA). Calculation shows at this current, electro-osmotic flow (*U*_eo_/*U* = −0.95) is on the same order of hydraulic flow (*U*_h_/*U* = 1.95) (see Supplementary Note 2). Supplementary Fig. 1 illustrates the temporal evolution of the interface under different negative currents, demonstrating the possibility of triggering finger growth for a hydrodynamically stable displacement in a large range of currents beyond a certain threshold (in this case, *I* = −2 mA). On the contrary, a positive current of the same magnitude does not change the stability of the interface (Fig. 3a, *I* = +2 mA), because the electro-osmotic flow is stabilizing in this case. Supplementary Fig. 2 illustrates the temporal evolution of the interface at different positive currents and the stabilizing effect can be observed at all currents.

For a hydrodynamically unstable displacement, *M* < 1, i.e. moving into the high viscosity phase, the interface is inherently unstable (Fig. 3b, *I* = 0 mA), but again subject to electrokinetic control. Just as before, a positive current drives a stabilizing electro-osmotic flow (apparent electrokinetic thinning). Supplementary Fig. 3 illustrates temporal evolution of interface at different positive currents, showing the increasing suppression of fingers with increasing current. At sufficiently large current, the interface motion transitions from unstable to stable (Fig. 3b, *I* = +8 mA). On the contrary, a negative current of the same magnitude makes the interface even more unstable (apparent electrokinetic thickening in water), as evidenced by the smaller wavelength of the finger pattern (Fig. 3b, *I* = −8 mA). The destabilizing effect of the electro-osmotic flow is also illustrated at other negative currents (Supplementary Fig. 4).

### Critical current scaling

**Fig. 4:**
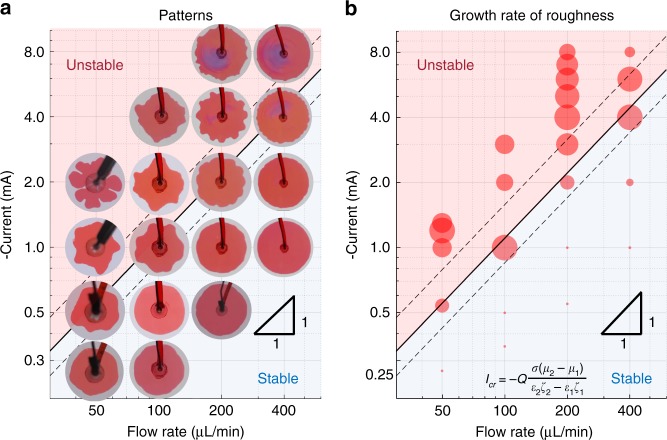
Stability diagram at different flow rates and currents (**M** = 1.98). **a** The patterns of the interfaces (*t* = 90 s). Clear fingers can be seen beyond a critical current for each flow rate. **b** Growth rate of the interfacial roughness. The growth rates of the interfacial roughness at different conditions are plotted as circles. The theoretical critical current is given by the inset equation and plotted as the solid line (*ζ*_2_ = *ζ*_w_ = −150 mV, *ζ*_1_ = *ζ*_o_ = 0 mV). Dashed lines are theory predictions with ±30% variation in *ζ*_w_. The predicted stable/unstable regions are shaded blue/red. The videos of the evolution of the interfaces are provided in Supplementary Movie 2

We note in this work we did not report systematic results in the hydrodynamically unstable case, mainly because our power source (max 10 mA, 2 kV) did not allow a systematic study at high currents and voltages. Nevertheless, we were able to perform systematic experiments in the hydrodynamically stable case, to highlight the role of electrokinetic phenomena. Typical interface shapes at different flow rates and negative currents are plotted in Fig. 4a. For each flow rate, no fingers are observed at small currents, but strong fingers can be observed beyond a critical current, which scales linearly with the flow rate. However, positive current of the same magnitude does not destabilize the interfaces (Supplementary Fig. 5), suggesting that both the direction and magnitude of the current are important.

To quantify the instability, we digitized the shape of the interface and did Fourier transform to extract each constituting mode (Supplementary Fig. 6). In principle, identifying the dominating mode, and verifying the dispersion relation predicted by the linear stability analysis would be a precise quantification of the instability mechanism. In some of our experiments, a dominant mode is easily identifiable (cf. Supplementary Fig. 6), but in most cases, experimental imperfections and initial perturbations make such an analysis quite difficult (cf. Supplementary Figs. 7 and 8). Instead, here we focus on testing the validity of the stability criteria in Eq. (3) by defining an “interfacial roughness” based on the root mean square (RMS) value of Fourier modes (see Quantitative Analysis of Interface Stability Section in Methods). Note that due to experimental noise, the interfacial roughness is non-zero even for a hydrodynamically stable case (*M* > 1) (Supplementary Fig. 5), but its magnitude does not grow in time. At small currents, the growth of interfacial roughness is also negligible. However, when the current is increased beyond a critical threshold, a steep rise in interfacial roughness is observed (Supplementary Fig. 9). We plot the growth rate of interfacial roughness at various flow rates and currents in Fig. 4b. Clearly, there is a boundary between the stable region (zero growth rate) and unstable region (large growth rate), which scales linearly with the flow rate as predicted by the theory (the inset equation of Fig. 4b).

Without adjusting any parameters, the theoretical prediction using the zeta potential value (*ζ*_w_ = −150 mV) of a water phase with similar composition (1 mM, pH = 10)^34^ is plotted as the solid line. The zeta potential of oil is small and poorly understood, and since the prediction is quite insensitive to its value (Supplementary Fig. 10), we set *ζ*_o_ = 0 mV (see Parameter Sensitivity Analysis Section in Methods). To guide the eye, theoretical predictions with ±30% variations of *ζ*_w_ (~2*V*_T_, where *V*_T_ ≈ 25 mV is the thermal voltage) are plotted as dashed lines to reflect uncertainty in the value of *ζ*_w_. The experimentally determined stability boundary falls well within the theoretically predicted region. Similar linear scaling of critical current vs flow rate is also observed at other viscosity ratios (Supplementary Fig. 11), and the predicted critical currents all agree well with the experiments within ±30% variation of *ζ*_w_.

### Stability phase diagram

**Fig. 5:**
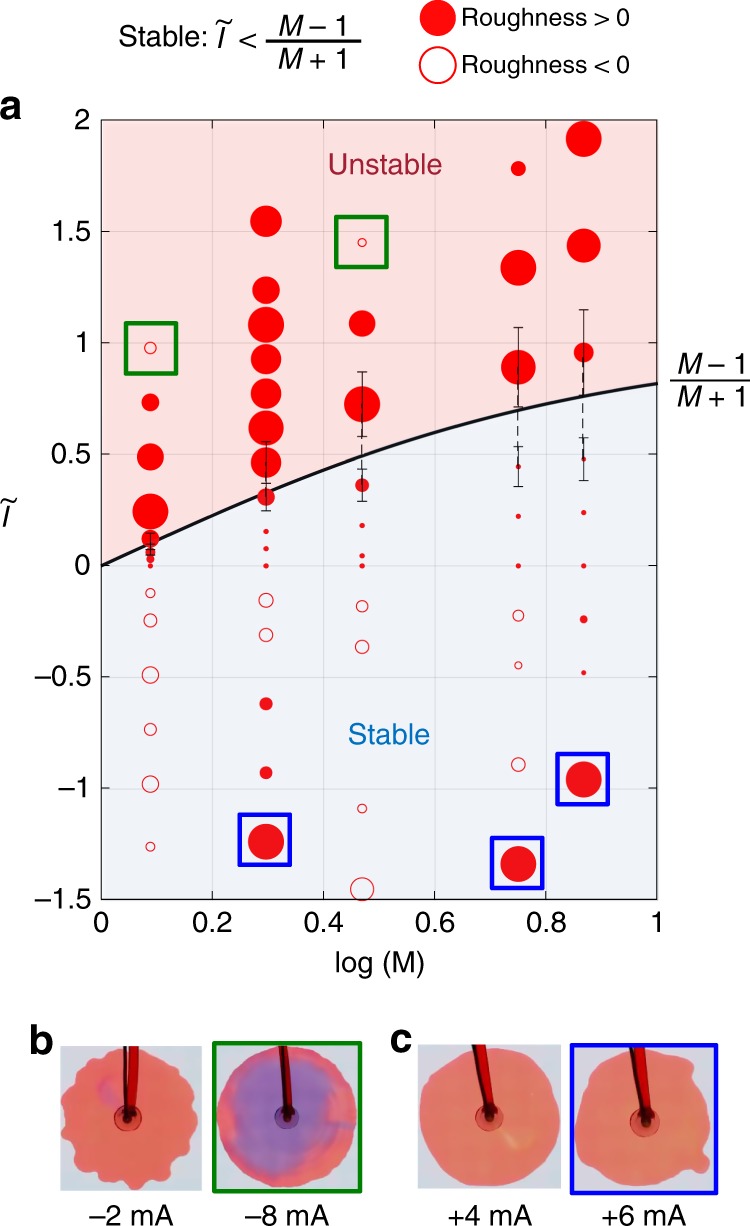
Dimensionless stability diagram. **a** Dimensionless stability diagram. The relative roughness at different currents with respect to purely hydrodynamical case (zero current) are plotted as circles, i.e. positive means less stable than hydrodynamical case (plotted as solid circles) and negative means more stable (plotted as open circles). Currents (*I*) are non-dimensionlized using Eq. (3). Note that a positive dimensional current (*I*) yields a negative dimensionless current $(\tilde I)$ since *ε*_w_*ζ*_w_ < *ε*_o_*ζ*_o_ in our experiments. For points near the theory line, error bars with ±30% variation in *ζ*_*w*_ are plotted for illustrating the uncertainty in water zeta potential. **b** Anomalous behavior at extreme negative currents. **c** Anomalous behavior at extreme positive currents

To examine the validity of stability condition (3), systematic experiments are performed at different viscosity ratios and currents. All experimental results are plotted in terms of the dimensionless current $(\tilde I)$ versus the viscosity ratio (M) (Fig. 5a). The solid line represents the theoretical prediction (cf. Eq. (3)), and the circles represent the interfacial roughness relative to the purely hydraulic case (zero current) for the same viscosity ratio. For small negative currents $(\tilde I_{{\mathrm{cr}}} > \tilde I > 0)$, interfacial roughness does not change appreciably, but a sharp increase is observed above the critical value $\tilde I > \tilde I_{{\mathrm{cr}}}$, suggesting the interface enters the unstable regime. In contrast, vanishing or negative interfacial roughness is observed with positive current $(\tilde I < 0)$, suggesting over-stabilization of the interface. The error bars in data points close to the boundary indicate ±30% variation in *ζ*_w_ to account for uncertainties in water zeta potential. Within the uncertainty, excellent agreement between the predicted and experimental stability boundaries is observed.

Although our theory holds over a wide range of conditions, some deviations are also observed under extreme currents. One anomalous case occurs at large negative currents (green square)(Fig. 5b), where fingers created at small currents (*I* = −2 mA) disappear at larger currents (*I* = −8 mA). This might be related to electrochemical reduction of the dye, as suggested by the substantial color change. We note, however, that reaction of the oil phase (1-octanol) is negligible (Damkohler number Da = 4.9 × 10^−3^, cf. Supplementary Fig. 12 and Supplementary Note 3). Another anomalous case happens at high positive currents (blue square), where the stable interface at intermediate current (*I* = +4 mA) become unstable at larger current (*I* = +6 mA) (Fig. 5c). We also note the fingering pattern is different compared to experiments at small negative currents (Fig. 3, *I* = −2 mA). In our set-up we use four metal spacers to separate two glass plates. In some of our experiments, the anomalous fingers show tendency to grow in the direction of one or more spacers (Supplementary Fig. 13).

## Discussion

Above we show the experimental evidence of active control of viscous fingering using electric field. Remarkably, both stable and unstable displacements are possible for any viscosity ratio, simply by adjusting the electrical current relative to the fluid flow rate. The mechanism can be understood by the concept of apparent “electrokinetic thickening/thinning”, as the pressure gradient must adjust to compensate for the opposing/assisting electro-osmotic flow, which leads to increased/reduced resistance for flow and transition of stability at critical current (electro-osmotic flow). This phenomenon is fundamentally different from the “electrohydrodynamic” control of leaky dielectric liquids by interfacial charge accumulation and Maxwell stress^35–37^, which may become important for electrolyte interfaces in larger electric fields^38,39^.

Many extensions of our discovery are possible. Beyond the canonical case of immiscible fluids, the idea of using electric fields to control viscous fingering is also applicable to miscible fluids. Analogous phenomena are also expected in porous media, due to similarity of the governing equations. In addition to the mechanism described here, electric fields and salinity effects could also impact viscous fingering by altering the surface wettability. Recent work indicates that the surface wettability can greatly impact interfacial patterns in both Hele-Shaw cells and porous media^40–43^. However, porous media flows are more complicated due to heterogeneity and anisotropy in the pore space, leading to capillary fingering^44,45^ and even dendritic patterns^46^ which are not considered in our theory and requires further investigation. The instability may also be controlled by actively modulating surface charge (or zeta potential) with additional electrodes, thus driving induced-charge electro-osmotic flows in each fluid domain^47^.

The results are consistent with the recently proposed theory of “electrokinetic” control of interfacial stability by bulk electro-osmotic flows in immiscible electrolytes^33^. However, we caution that our theory does not capture nonlinear effects that might be relevant at higher currents and cause further anomalies. First, in our analysis we have ignored electrohydrodynamic (EHD) effects, related to formation of unscreened charge and large Maxwell stresses at the liquid/liquid interface^35,36,48^, which tend to destabilize the interface. However, electric fields in our experiments (~0.3 kV cm^−1^) are much smaller than the typical range where EHD dominates (>10 kV cm^−1^). Second, there may also be EDL formation at the interface, which can cause EHD-like flows^32,39^ and reduce surface tension through electrocapillary effect^49,50^, thereby shifting the most unstable wavenumber to larger values. It is important to note that both EHD and electrocapillary effects depend on Maxwell stress, scaling as *τ*_M_~*E*^2^, and thus are destabilizing at any current, either positive or negative. In contrast, linear electrokinetic phenomena offer the possibility of switching the stability of the interface, simply by reversing the direction of the electric field (Supplementary Figs. 14 and 15).

Our theory is based on a depth-averaged model of interfacial displacement. Despite its popularity, recent studies suggest that three-dimensional effects and wetting films might dramatically affect the patterns^41,43,51^. Since electro-osmotic flows are nearly uniform across the gap, flow reversal is possible close to the surface (see Fig. 1c). This might impact the contact-line motion. We have also ignored the possibility of wetting films in our analysis but this is justified since the capillary number in our experiments is typically around Ca~10^−5^ (see Quantitative Analysis of Interface Stability Section in Methods), considerably below the critical value for a wetting transition^52,53^. Nevertheless, more sophisticated models, three-dimensional simulations, and careful experiments are required to assess the validity of these assumptions, which are beyond the scope of the current work.

In conclusion, we have reported the first experimental evidence for active control of viscous fingering via external electric field by introducing electro-osmotic flow into the system, and provided a simple physical picture to explain the experimental observations. Both suppression and promotion of the instability are possible by properly tuning the direction and strength of electric field in a Hele-Shaw cell. The possibility of active control of viscous fingering using electric fields may find diverse applications. Electrokinetic suppression of the fingering instability would enhance secondary oil recovery, while promoting it could improve mixing efficiency in microfluidics and porous media, as in CO_2_ sequestration. Our results are also relevant for devices involving the “interface between two immiscible electrolyte solutions” (ITIES)^50,54–56^, where direct electrokinetic control of interfacial patterns for electro-wetting^57^ or electro-tunable optics^58^ might lead to novel applications in confined geometries. More generally, this work exemplifies the rich physics of “multi-field” driven interfacial dynamics^59,60^, which has recently shown promising results in controlling morphological instabilities in electro-deposition^61,62^. It is conceivable that similar electrokinetic strategies could be used to enable electro-tunable adhesion^63,64^, pattern soft materials^17^, control drug delivery in layered bodily tissues^65^, or enhance mixing in microfluidic devices^16^.

## Methods

### Experimental conditions

To construct the Hele-Shaw cell, two glass disks (5″ in diameter and 0.25″ thick) are stacked together separated by four stainless steel spacers (gap *h* = 200 μm). The center electrode is housed inside a cone glued on the top glass, which also functions as a reservoir to store any gases produced by the reduction of octanol or water so that they do not interfere the two-liquid-phase flow. The Hele-Shaw cell (stacked glass plates) is contained in a shallow round can made of acrylic. During the experiment, the water coming out from the Hele-Shaw cell will enter the annulus-shaped reservoir between the inner wall of the can and the Hele-Shaw cell. After each experiment, we drain the fluid in the reservoir.

1-octanol is used as the oil for its immiscibility with water (solubility in water 0.3 g L^−1^), modest surface tension (37 mN m^−1^) and viscosity (7.36 mPa s), and slight polarity (*ε*_r_ = 10.3). Tetrabutyl-ammonium chloride (TBACl) is added into the oil to tune its conductivity. Glycerol is added at varied wt% into water to tune its viscosity. The pH of the water phase is adjusted to 10 by adding NaOH, and KCl is used to tune its conductivity. The conductivities of both water and oil are maintained the same. The concentration of KCl is ≤2 mM. The oil phase is dyed red by 0.1 wt% oil red.

Typical experimental conditions: Fig. 3a, Red: octanol − 0.75 M TBACl − 0.1% oil red, *σ*_i_ = 155 uS cm^−1^, *μ*_i_ = 7.36 mPa s, *ε*_ri_ = 10.3. Light blue: water/glycerol (60/40 w/w), pH = 10, *σ*_o_ = 155 uS cm^−1^, *μ*_*o*_ = 3.72 mPa s, *ε*_ro_ = 68.8. Flow rate: *Q* = 200 uL min^−1^, *t* = 120 s. Figure 3b, Red: same as Fig. 3a. Light blue: water/glycerol (15/85 w/w), pH = 10, *σ*_*o*_ = 155 uS cm^−1^, *μ*_o_ = 109 mPa s, *ε*_ro_ = 49.1. Flow rate: *Q* = 400 uL min^−1^, *t*  = 60 s.

Under typical experimental conditions, the buildup of reaction products in bulk solution is negligible. The evolution of the interface is recorded with a digital camera and then digitized into a 2D polar plot for quantitative analysis. The direction of the flow is always from the center to the edge and positive current is defined to have the same direction as the flow. The oil phase is slightly more wetting on the glass than the water phase, with a contact angle of about 95 degrees.

### Quantitative analysis of interface stability

The obtained image of the interface was first digitized into a polar plot, *R*(*θ*), then Fourier Transform was performed to calculate the roughness of the interface according to:

4 $${\mathrm{Roughness}} = \sqrt {2\mathop {\sum}\limits_{n = 1}^\infty {\frac{{C_n^2}}{{C_0^2}}} } ,$$

5 $$C_n = \frac{1}{{2\pi }}\mathop {\int}\limits_{ - \pi }^\pi R (\theta )e^{ - in\theta }d\theta ,$$

The dimensionless Fourier coefficients *C*_*n*_ is the relative length of each perturbation mode with respect to the average radius of the pattern (base mode *C*_0_), which measures the relative strength of each individual mode (Supplementary Figs. 6–8). When fingers are strong and symmetric, dominating mode can be identified. However, most of the time there can be several modes with similar strength because of non-symmetric or weak fingers, which makes the identification of dominating mode arbitrary. For this reason we do not report the analysis of individual mode, e.g. growth rate of dominating mode for obtaining the dispersion relation. Instead, we use roughness to measure the overall instability of the pattern.

The roughness of two typical experiments are plotted in Supplementary Fig. 9. Roughness does not change appreciably for small or zero currents. Beyond the critical current, roughness rapidly increases as labeled by the red arrow in the figure. During the experiment, roughness reaches its peak value then levels off or drops. Note the roughness or its growth rate are nonzero at zero current. For a hydrodynamically stable displacement, some instability can still be observed (nonzero roughness and nonzero growth), see Supplementary Fig. 5 for the non-circular patterns and Supplementary Fig. 9 for the quantitative measurement. We calculate the capillary number for Fig. 3a at *t* = 120 s (*R* = 2.52 cm) for understanding this instability

6 $${\mathrm{Ca}} = \frac{{U\mu }}{\gamma } = \frac{{\frac{{200\,{\mathrm{uL}}\,{\mathrm{min}}^{ - 1}}}{{2\pi \times 2.52\,{\mathrm{cm}} \times 200\,{\mathrm{um}}}} \times 3.72\,{\mathrm{mPa}}\,{\mathrm{s}}}}{{37\,{\mathrm{mN}}\,{\mathrm{m}}^{ - 1}}} \approx 10^{ - 5}$$

The very wide bump on the patterns observed at zero currents in Fig. 3a could be related to capillary forces due to non-uniformity in the gap thickness. These patterns gradually disappear as we increase the flow rate (increasing Ca) (see Supplementary Fig. 5). To exclude this effect when plotting the roughness at different currents, only the offset from zero current is plotted.

### Parameter sensitivity analysis

In Fig. 4b we plot the critical current,

7 $$I_{{\mathrm{cr}}} = - Q\frac{{\sigma \left( {\mu _{\mathrm{w}} - \mu _{\mathrm{o}}} \right)}}{{\varepsilon _{\mathrm{w}}\zeta _{\mathrm{w}} - \varepsilon _{\mathrm{o}}\zeta _{\mathrm{o}}}},$$

to compare with experiment results. In our experiments, we are able to control or measure all parameters accurately except for *ζ*_w_ and *ζ*_o_, which characterize the surface charge of glass in the water or oil phase, respectively. We tried to characterize *ζ*_w_ and *ζ*_o_ through streaming potential measurements in a rectangular channel. However, the experiment was not successful because the streaming potential did not reach equilibrium even after 20 h. For this reason, we use the experiment results reported for water/alcohol mixture of the same concentration and pH, i.e. *ζ*_w_ = −150 mV^34^. For the oil phase, we could not find reliable data in the literature. However, since the oil phase is considerably less polar (dielectric constant of 10.3 vs. 78.2 for water), we expect the surface potential to be small and therefore |*ε*_o_*ζ*_o_|≪|*ε*_w_*ζ*_w_|. The sensitivity of the equation on the value of *ζ*_w_ and *ζ*_o_ are given in Supplementary Fig. 10. As can be seen, the position of the critical current is dominated by *ζ*_w_ and is almost independent on *ζ*_o_. Therefore, we have consistently used *ζ*_o_ = 0 mV when plotting theory predictions (solid lines).

## Supplementary information

supplementary information

peer review file

Description of Additional Supplementary Files

supplementary movie 1

supplementary movie 2

## Data Availability

The data supporting the findings of this study are available within the paper and its Supplementary files, and are available from the corresponding author upon request.

## Code availability

The MATLAB code used in processing the experiment data is available from the corresponding author upon request.
