# Supplementary material for: Active control of viscous fingering using electric fields
Source: Nat Commun. 2019 Sep 5;10:4002. doi: 10.1038/s41467-019-11939-7 (PMC6728344; doi:10.1038/s41467-019-11939-7)
Supplement: Supplementary file 1 — supplementary information [file 41467_2019_11939_MOESM1_ESM.pdf]

Supplementary Information:
Active Control of Viscous Fingering Using Electric Fields

Tao Gao,[1,*] Mohammad Mirzadeh,[1,*] Peng Bai,[1,†]
Kameron M. Conforti,[1] and Martin Z. Bazant[1,2,‡]

[1] *Department of Chemical Engineering, Massachusetts Institute of Technology, MA 02139.*

[2] *Department of Mathematics, Massachusetts Institute of Technology, MA 02139.*

* T.G. and M.M. contributed equally to this work.

† Current address: Department of Energy, Environmental, and Chemical Engineering, Washington University in St. Louis, MO 63130.

‡ Corresponding author: bazant@mit.edu

I. SUPPLEMENTARY FIGURES

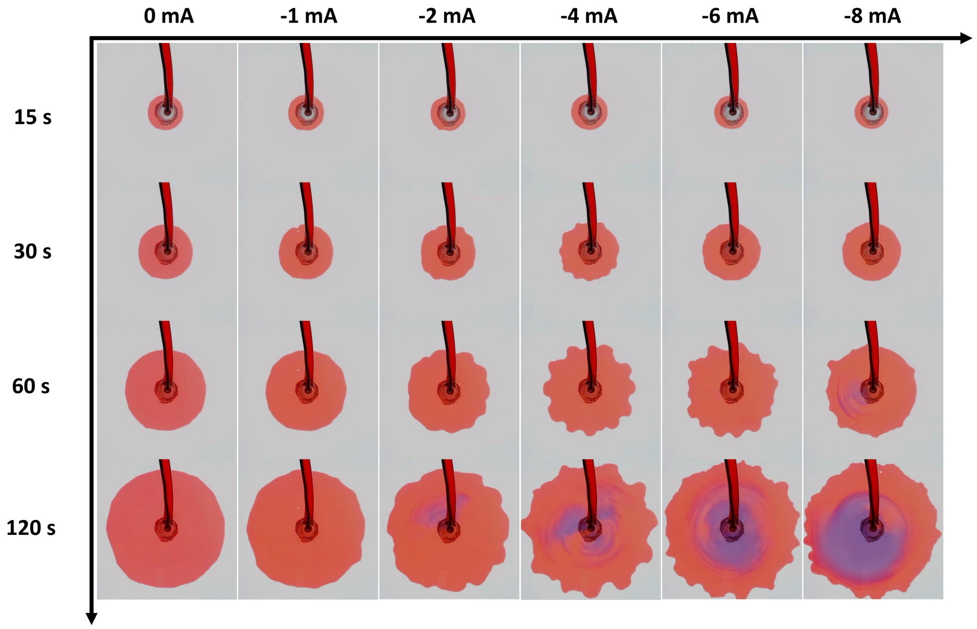

Supplementary Figure 1. **Patterns of a hydrodynamically stable displacement at different negative currents.** ($M = 1.98 > 1$). Clear fingers can be seen when the magnitude of current is beyond a certain threshold (in this case, $I = -2$ mA). Strong and symmetric fingers can be observed at intermediate currents (-2, -4, -6 mA) and below 60 s. However, at higher currents or longer times, an asymmetric growth is observed. Reduction reaction happens at the center electrode, which reduces the dye from red to purple.

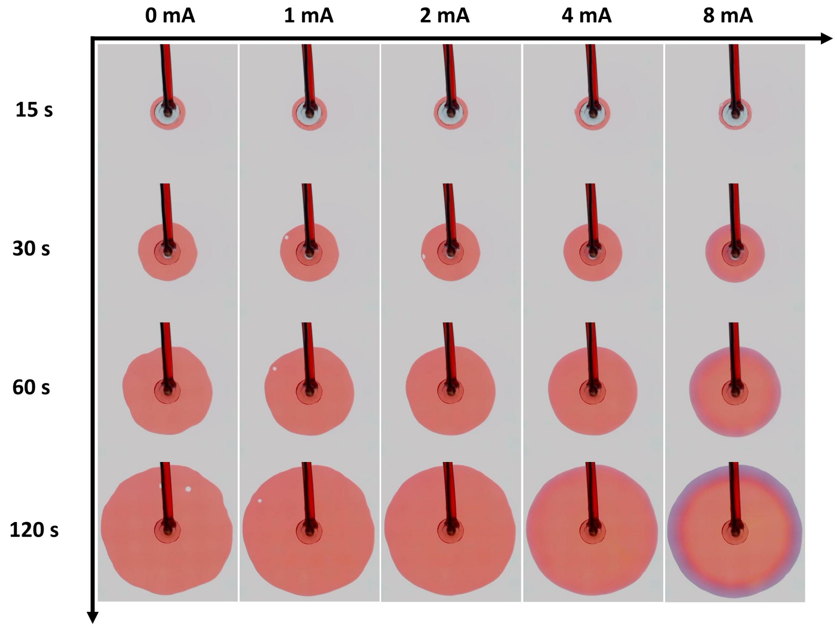

Supplementary Figure 2. **Patterns of a hydrodynamically stable displacement at different positive currents.** ($M = 1.23 > 1$). For the hydrodynamically stable displacement, we do observe certain instability at zero current, suggested by the asymmetry of the pattern. This might be related to capillary forces due to non-uniformity of gap thickness. Nevertheless, suppression of the instability occurs when positive currents is applied, as suggested by the more symmetric shape of the interface as well as a decrease in the roughness measure. At high current, dye could be reduced at the interface, indicating the flux of ions is not sufficient to sustain the current.

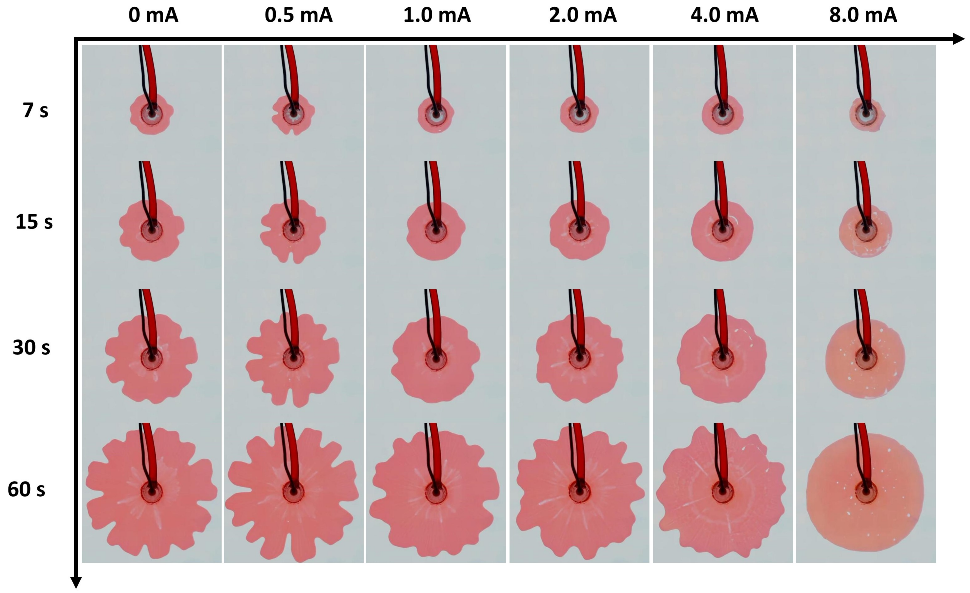

Supplementary Figure 3. **Patterns of a hydrodynamically unstable displacement at different positive currents.** ($M = 0.067 < 1$). Suppression of fingers is observed when positive current is applied (shortening of fingers), but full suppression is only achieved at a sufficiently high current (in this case, $I = 8$ mA).

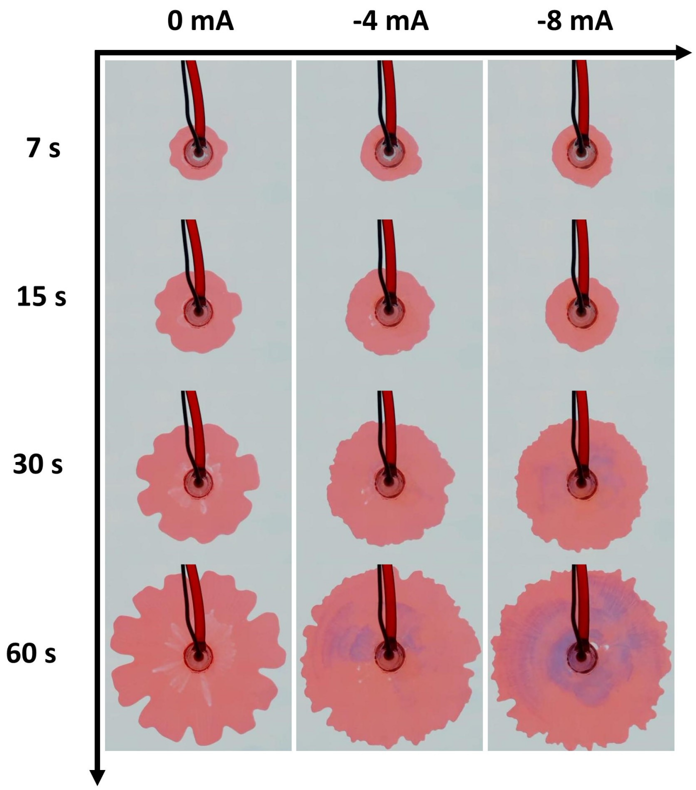

Supplementary Figure 4. **Patterns of a hydrodynamically unstable displacement at different negative currents.** ($M = 0.067 < 1$). Negative current renders the interface more unstable, as evident from shortening of instability wavelength.

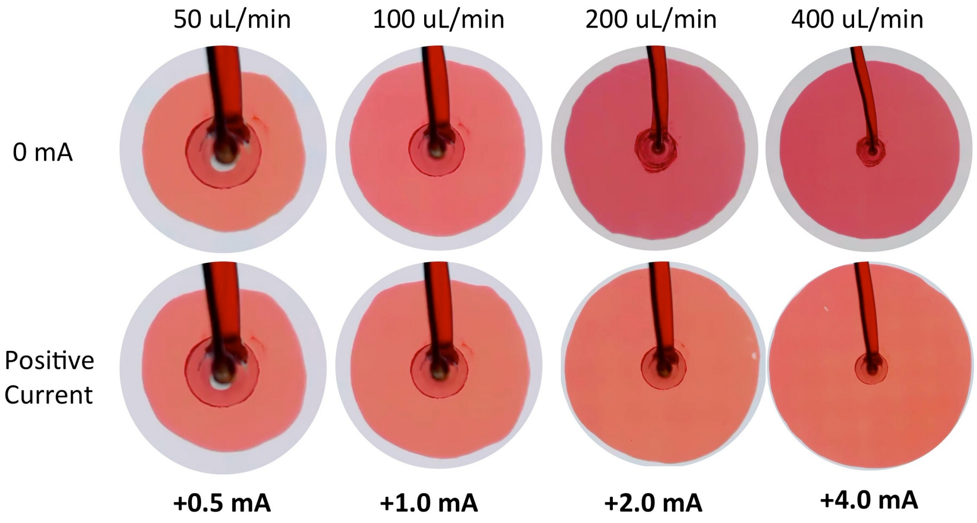

Supplementary Figure 5. **Patterns at positive currents and zero current for different flow rates.** The magnitude of the positive current is the same as the negative current that just destabilizes the interface in Figure 4a.

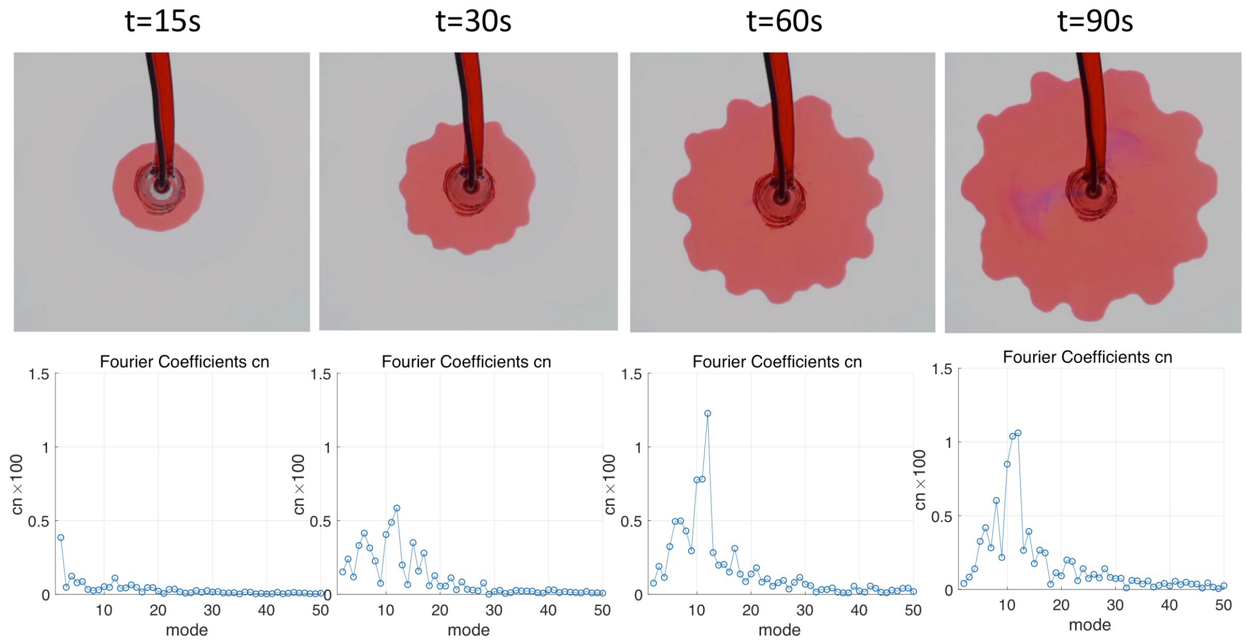

Supplementary Figure 6. **Fourier Transform of the interface of oil pushing water-glycerol 60/40 at -4 mA.** The Fourier coefficients (C_n) evaluates the relative length of each perturbation mode with respect to the average radius of the pattern (C_0). In this figure, the pattern is quite symmetric so the dominating mode (in this case, C_{12}) can be readily identified. For most experiments, however, the pattern is not as symmetric which leads to the co-existence of many strong modes.

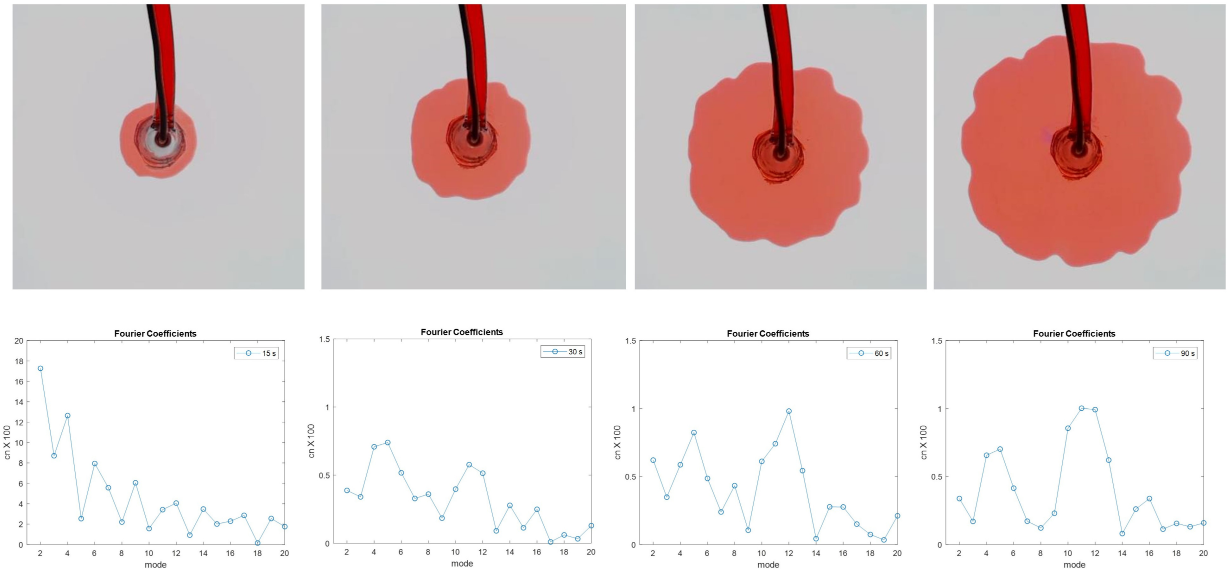

Supplementary Figure 7. **Fourier Transform of the interface of oil pushing water-glycerol 60/40 at -3 mA.** The dominant mode can not be identified because there are several modes having similar strength.

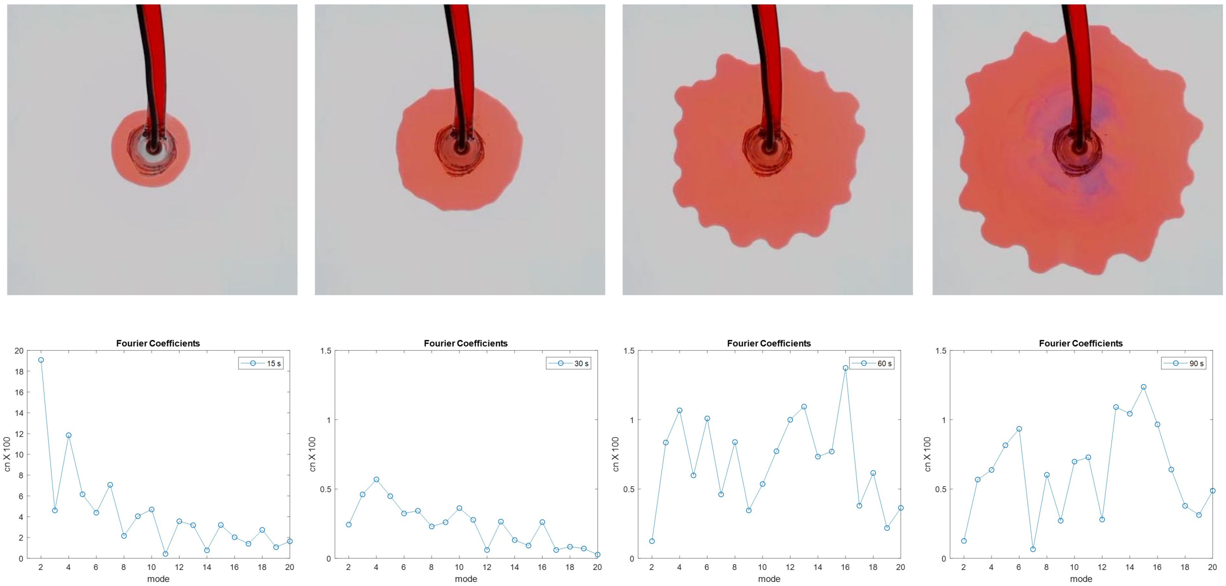

Supplementary Figure 8. **Fourier Transform of the interface of oil pushing water-glycerol 60/40 at -6 mA.** The dominant mode can not be identified because there are several modes having similar strength.

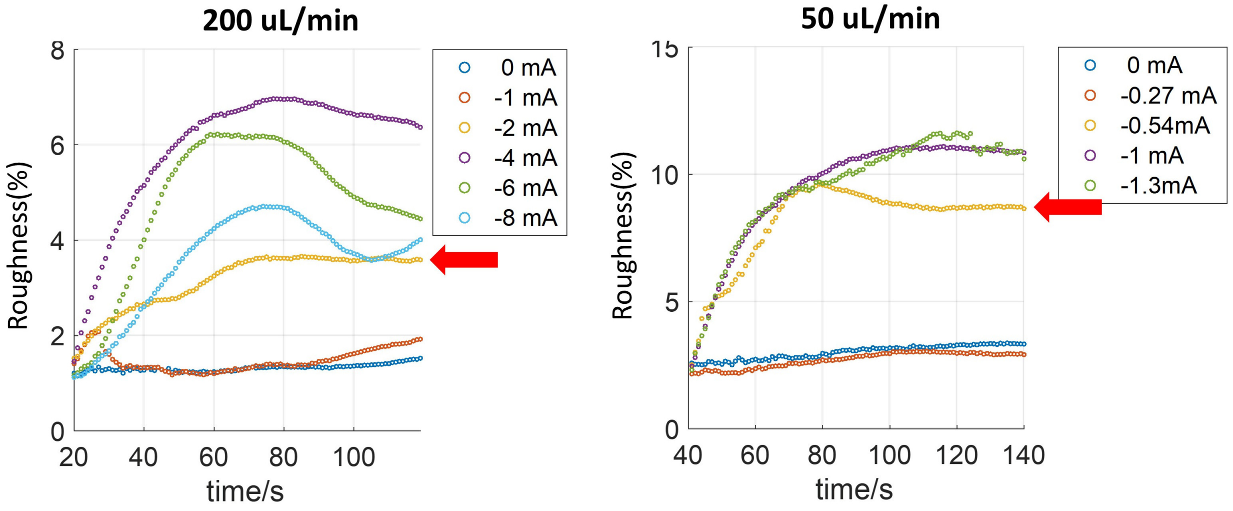

Supplementary Figure 9. **Roughness vs time.** At small or zero currents, the roughness is nearly constant. However, when the current is strong enough ($I = -2$ mA for $Q = 200$ uL min^{-1} and $I = -0.54$ mA for $Q = 50$ uL min^{-1}, indicated by the red arrows), the roughness increases considerably. The growth rate of roughness is defined to be the slope of the linearly increasing region. The peak roughness is the maximum roughness on the curve.

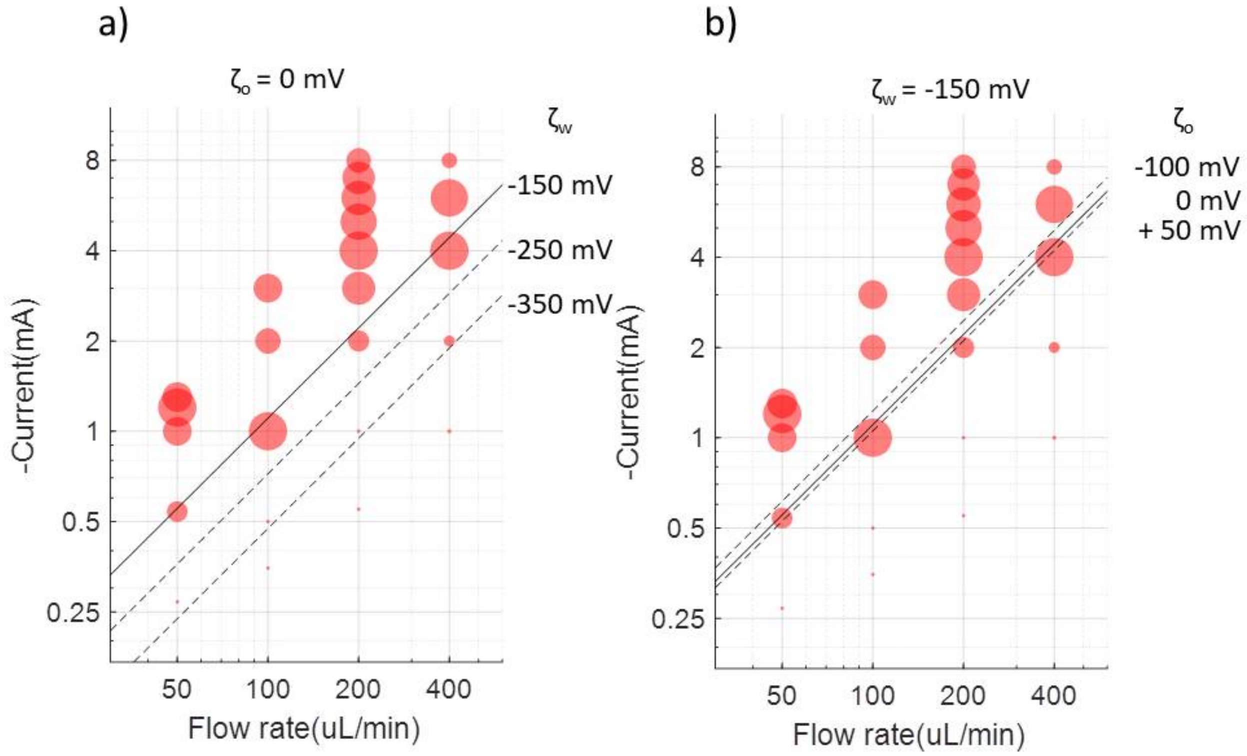

Supplementary Figure 10. **Parameter sensitivity analysis**. **(A)** $\zeta_o = 0$ mV and ζ_w varies; **(B)** $\zeta_w = -150$ mV and ζ_o varies. The theory prediction is not sensitive to ζ_o.

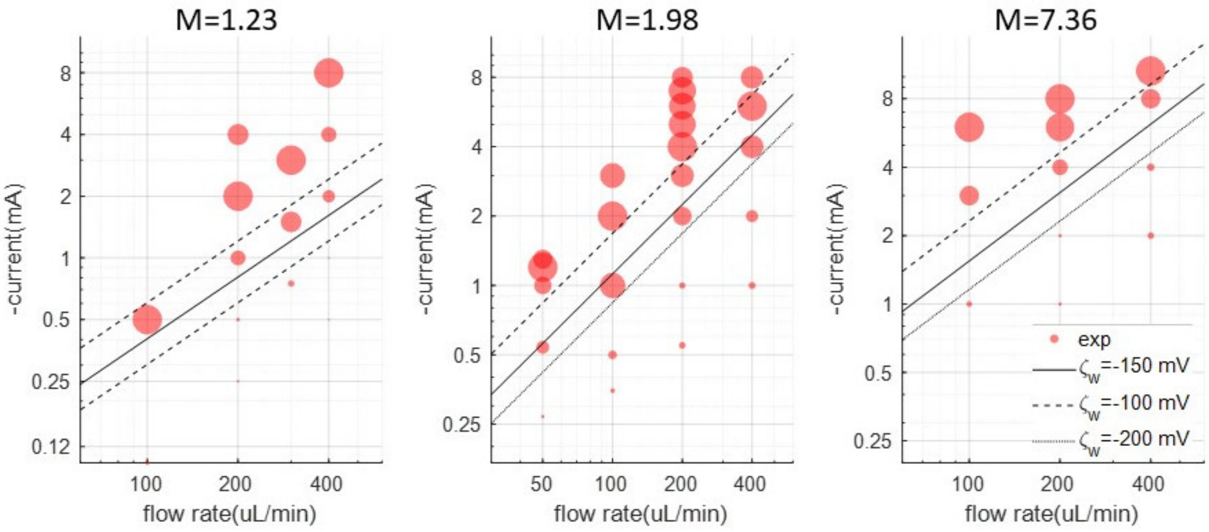

Supplementary Figure 11. **Roughness measure at different viscosity ratios**. The peak roughness at each flow rate and current is plotted in each figure. Roughness at each flow rate is normalized by the maximum roughness at this flow rate to make results at different currents comparable. At all viscosity ratios, the boundary of stability scales linearly with flow rate (notice both axes have logarithmic spacing). Theory predictions at different ζ_w and $\zeta_o = 0$ mV are plotted for comparison.

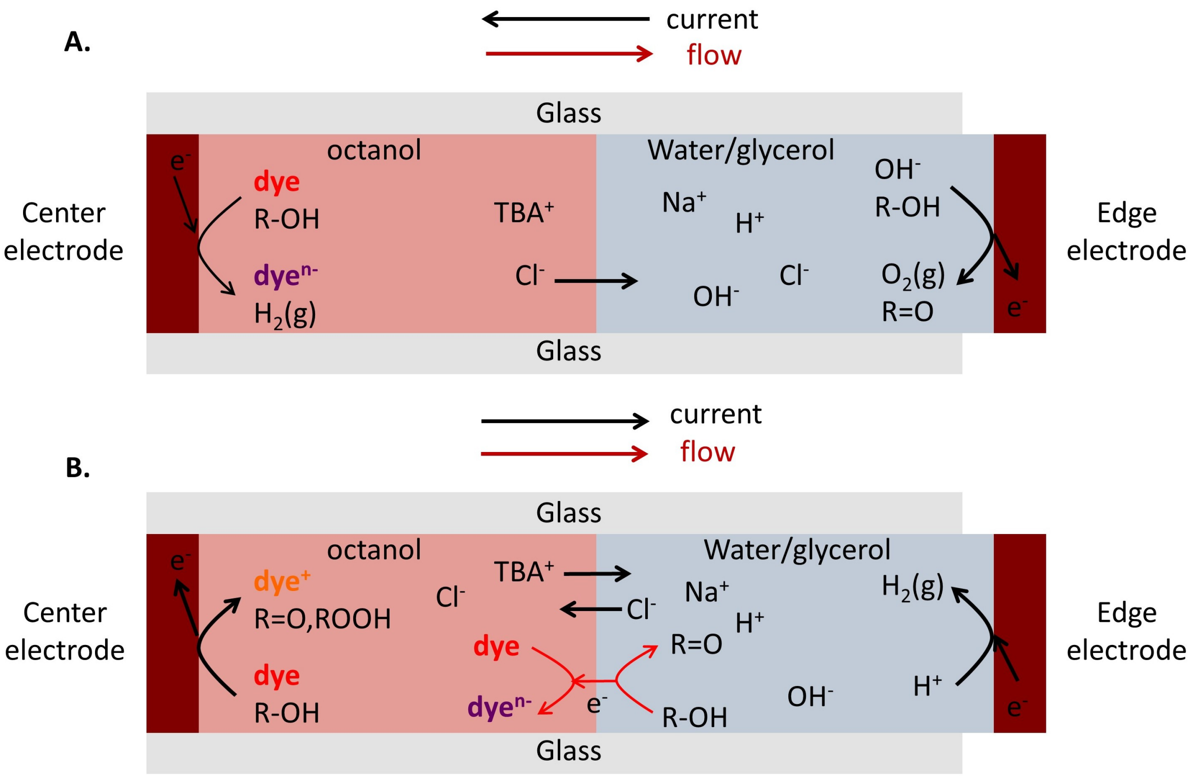

Supplementary Figure 12. **Electrochemical reactions at the interfaces.** The cross-section of the cell is shown with center electrode at left and edge electrode at right. When an external electric field is applied across the two electrodes, electrochemical reactions occur at the electrode/electrolyte interface to sustain the current while ions migrate in electrolyte bulk to conduct the current. At the octanol/water-glycerol interface, only TBA^+ and Cl^- can move across the boundary, while Na^+, K^+, H^+, and OH^- can not. **(A)** Negative current. Reduction of dye (color change to purple) and reduction of octanol occur at the center electrode, while oxidation of water and glycerol occur at the edge electrode. At the interface between octanol/water-glycerol, Cl^- migrates across the boundary to sustain the current. **(B)** Positive current. Oxidation of dye (color change to orange) and octanol occur at the center electrode, while reduction of water occur at the edge electrode. At the interface between octanol/water-glycerol, both TBA^+ and Cl^- migrate across the boundary to sustain the current. At large currents, reduction of dye can also happen at the octanol side of the interface to maintain the current, while on the water-glycerol side, oxidation of glycerol happens to provide the electrons (labelled in red). The reaction of octanol is negligible, but the reaction of dye can be significant at high currents. See Fig. 5 and relevant discussions.

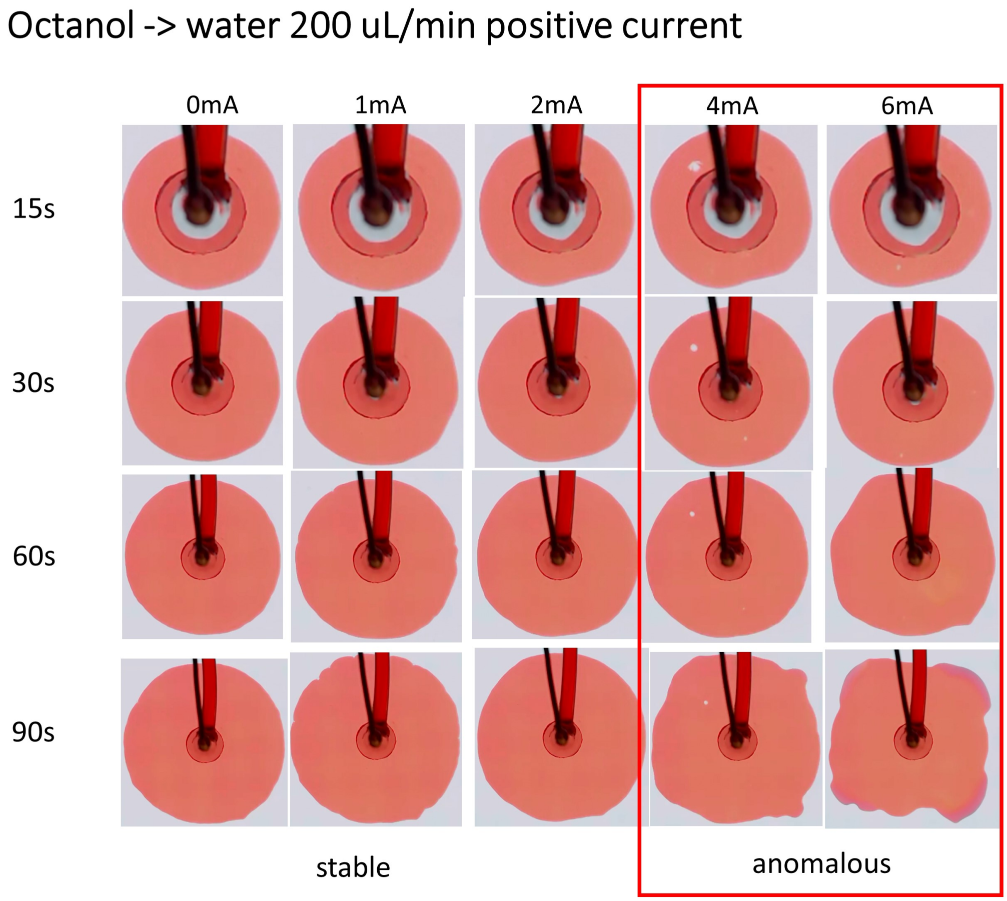

Supplementary Figure 13. Interfaces for oil pushing water at different positive currents

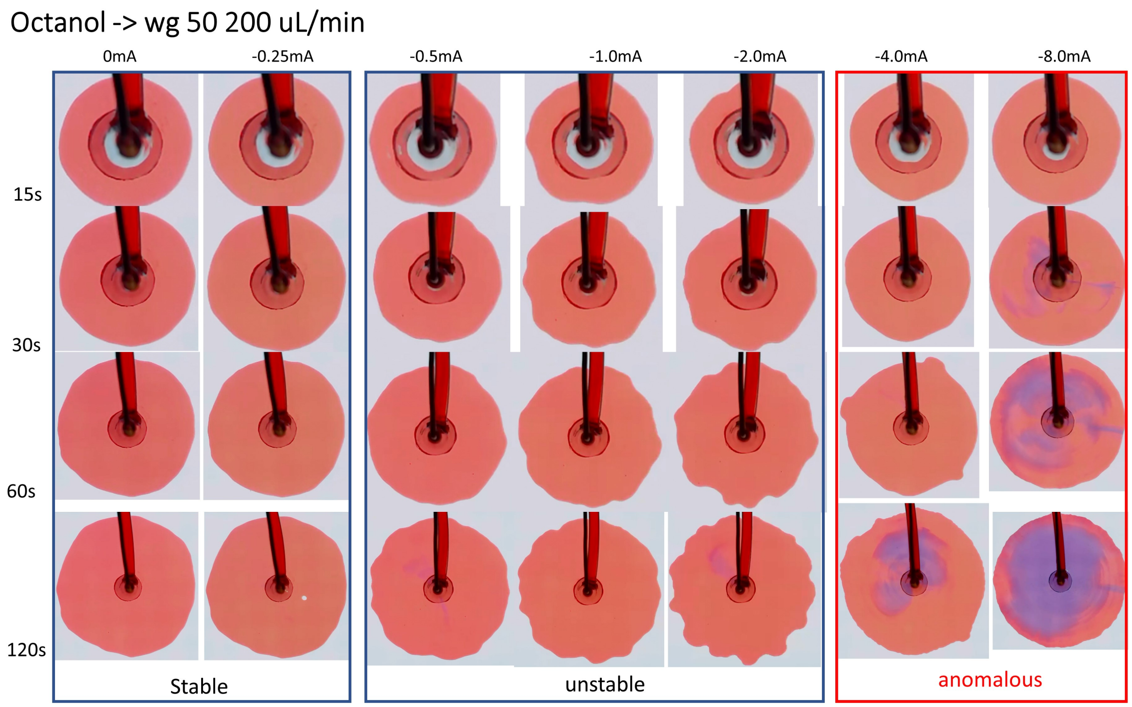

Supplementary Figure 14. Interfaces for oil pushing water-glycerol 50/50 at different negative currents

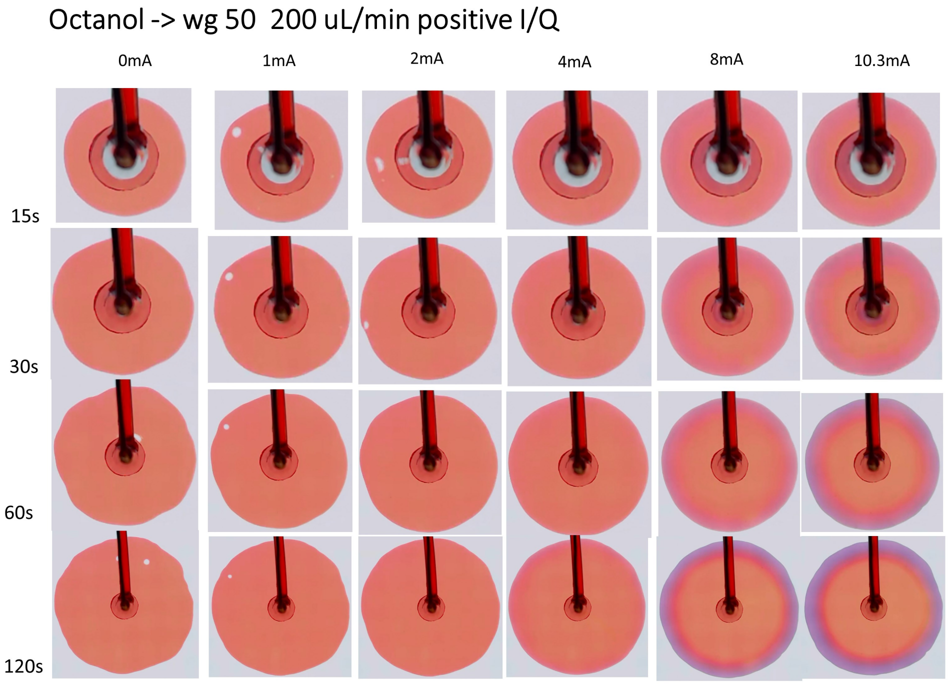

Supplementary Figure 15. Interfaces for oil pushing water-glycerol 50/50 at different positive currents

II. SUPPLEMENTARY NOTES

Supplementary Note 1: Theory

The theory behind our experiments, originally proposed by Mirzadeh and Bazant [1], is briefly mentioned here for the sake of completeness. The interface motion is described by extending the Darcy's equation to include coupled electrokinetic phenomena. Since the cell gap, h, is very small ($h \ll R_o$, where R_o is the outer radius of the cell) the governing equations are written in terms of generalized depth-averaged fluxes. The total velocity in each phase is the sum of hydraulic and electro-osmotic parts,

$$\mathbf{u} = \mathbf{u}_{\mathrm{h}} + \mathbf{u}_{\mathrm{eo}} = -K_{\mathrm{h}}\nabla p - K_{\mathrm{eo}}\nabla\phi, \tag{1}$$

where p is the pressure, ϕ is the electrostatic potential, and $K_{\mathrm{h}} = h^2/12\mu$ and $K_{\mathrm{eo}} = -\varepsilon\zeta/\mu$ are the hydraulic and electro-osmotic mobilities of the cell, respectively. The material coefficients, μ, ε, and ζ are the viscosity, permittivity, and the surface potential in each phase, respectively. Similarly, the total current is the sum of streaming and Ohmic parts,

$$\mathbf{i} = \mathbf{i}_{\mathrm{sc}} + \mathbf{i}_{\mathrm{e}} = -K_{\mathrm{eo}}\nabla p - K_{\mathrm{e}}\nabla\phi, \tag{2}$$

where $K_{\mathrm{e}} = \sigma$ is the electrical conductivity of each phase. Along with the statement of conservation of mass and charge, these equations may be written in the following generalized Darcy form:

$$\mathbf{F} = -\mathbb{K}\nabla\mathbf{\Phi}, \quad \nabla\cdot\mathbf{F} = \mathbf{0}, \quad \mathbb{K} = \begin{pmatrix} K_{\mathrm{h}} & K_{\mathrm{eo}} \\ K_{\mathrm{eo}} & K_{\mathrm{e}} \end{pmatrix}, \tag{3}$$

where $\mathbf{F} = (\mathbf{u}, \mathbf{i})^{\mathsf{T}}$ and $\mathbf{\Phi} = (p, \phi)^{\mathsf{T}}$ are the generalized fluxes and driving forces, respectively, and $\mathbb{K}$ is the so-called electrokinetic coupling tensor. The linear relationship between fluxes and driving forces is reminiscent of non-equilibrium thermodynamics and can be derived directly from Stokes and Poisson-Nernst-Planck equations [2]. At the interface, the normal component of fluxes and electric potential are continuous and the pressure jump satisfies the Young-Laplace equation,

$$[\![\hat{\mathbf{n}}\cdot\mathbf{F}]\!] = \mathbf{0}, \quad [\![\mathbf{\Phi}]\!] = (\gamma\kappa, 0)^{\mathsf{T}}, \tag{4}$$

where $[\![a]\!] = a_2 - a_1$ denotes the jump of variable 'a' across the interface and γ and κ are the interfacial tension and in-plane curvature of the interface. Finally the interface motion is as:

$$\frac{\mathrm{d}\mathbf{x}}{\mathrm{d}t} = (\hat{\mathbf{n}}\cdot\mathbf{u})\hat{\mathbf{n}}. \tag{5}$$

The interface motion is analyzed using the standard Linear Stability Analysis [3, 4]. We consider a circular interface of radius R, superimposed by small azimuthal perturbations of the form $\delta r \sim \exp(ik\theta+\omega t)$ and seek solutions of the form $\mathbf{\Phi} = \mathbf{\Phi}_0 + \epsilon\mathbf{\Phi}_1$ where $\epsilon \ll 1$. Here, k is the wavenumber and ω is the growth rate. Assuming constant flow rate, Q, and electrical current, I, at the center yields the base state solution:

$$\mathbf{\Phi}_{j,0} = -\mathbb{K}_j^{-1} \cdot \mathbf{F}_0 \log \frac{r}{R} + (\gamma/R, 0)^{\mathsf{T}} . \tag{6}$$

Here the subscript 'j' represents the two fluids ($j = 1$ for inside and $j = 2$ for outside), $\mathbb{K}_j^{-1}$ is the inverse of the conductivity matrix in fluid 'j', and $\mathbf{F}_0 = (U, J)^{\mathsf{T}} = (Q, I)^{\mathsf{T}}/2\pi Rh$ are the instantaneous fluid velocity and current density at the interface. Similarly the solution to the perturbation field is:

$$\mathbf{\Phi}_{j,1} = \mathbf{A}_j \exp(ik\theta + \omega t) \left(\frac{r}{R}\right)^{m_j}, \quad m_j = \begin{cases} k, & j = 1 \\ -k, & j = 2 \end{cases} . \tag{7}$$

The constants $\mathbf{A}_j$ are determined by applying the jump boundary conditions in supplementary equation (4):

$$\mathbf{A}_1 = \frac{\gamma(k^2-1)}{R^2} (\mathbb{K}_1 + \mathbb{K}_2)^{-1} \mathbb{K}_2 \cdot (1, 0)^{\mathsf{T}} + (\mathbb{K}_1 + \mathbb{K}_2)^{-1} \mathbb{K}_2 \left(\mathbb{K}_1^{-1} - \mathbb{K}_2^{-1}\right) \cdot \mathbf{F}_0, \tag{8}$$

$$\mathbf{A}_2 = -\mathbb{K}_2^{-1} \mathbb{K}_1 \cdot \mathbf{A}_1. \tag{9}$$

The growth rate, ω, is found by enforcing the compatibility condition (supplementary equation (5)):

$$U + \epsilon\omega \exp(ik\theta + \omega t) = -\mathbb{K}_1 \frac{\partial}{\partial r} (\mathbf{\Phi}_0 + \epsilon \mathbf{\Phi}_1) \cdot (1, 0)^{\mathsf{T}}, \tag{10}$$

which yields:

$$\omega = -\frac{U}{R} + \frac{k}{R}(1, 0) \cdot \left(\mathbb{B} \cdot \mathbf{F}_0 - \frac{(k^2-1)}{R^2} \mathbb{C} \cdot (1, 0)^{\mathsf{T}}\right), \tag{11}$$

$$\mathbb{B} = \mathbb{K}_1 (\mathbb{K}_1 + \mathbb{K}_2)^{-1} \mathbb{K}_2 \left(\mathbb{K}_2^{-1} - \mathbb{K}_1^{-1}\right), \tag{12}$$

$$\mathbb{C} = \mathbb{K}_1 (\mathbb{K}_1 + \mathbb{K}_2)^{-1} \mathbb{K}_2. \tag{13}$$

For our Hele-Shaw experiments, the electrokinetic coupling coefficient, $\alpha = K_{\mathrm{eo}}^2/K_{\mathrm{h}}K_{\mathrm{e}}$, is exceedingly small, $\alpha \sim \mathcal{O}(10^{-11} - 10^{-8})$, and the supplementary equations (11)-(13) greatly simplify, yielding the following dispersion relation:

$$\omega(k) = -\frac{U}{R} + \frac{k}{R}\left(U\frac{\mu_2 - \mu_1}{\mu_2 + \mu_1} + 2J\frac{\varepsilon_2\zeta_2 - \varepsilon_1\zeta_1}{(\sigma_2 + \sigma_1)(\mu_2 + \mu_1)} - \frac{h^2\gamma(k^2-1)}{12R^2(\mu_2 + \mu_1)}\right), \tag{14}$$

where U and J are the unperturbed interface velocity and current density, respectively. We conduct our experiments under constant flow rate, Q, and total electric current, I, which are related to velocity and current density via $U = Q/2\pi Rh$ and $J = I/2\pi Rh$. From supplementary equation (14), stability is guaranteed for all k if:

$$Q\left(\mu_2 - \mu_1\right) + 2I\frac{\varepsilon_2\zeta_2 - \varepsilon_1\zeta_1}{\sigma_2 + \sigma_1} < 0, \tag{15}$$

which is a more general form of equation (2) in the main text. Supplementary equation (15), may be expressed in non-dimensional form by introducing the non-dimensional control parameter,

$$\tilde{I} = \frac{2I}{Q}\frac{(\varepsilon_2\zeta_2 - \varepsilon_1\zeta_1)}{(\sigma_1 + \sigma_2)(\mu_1 + \mu_2)}, \tag{16}$$

where $M = \mu_1/\mu_2$ is the viscosity ratio. The stability condition then becomes:

$$\text{Stable:} \quad \tilde{I} < \tilde{I}_{\text{cr}} = \frac{M-1}{M+1}. \tag{17}$$

When the stability condition (supplementary equation (17)) is violated, the most dangerous wavenumber is given by:

$$\frac{\mathrm{d}\omega}{\mathrm{d}k} = 0 \Rightarrow k_m = \sqrt{\frac{1}{3} + 4\epsilon^{-2}\mathrm{Ca}(\tilde{I} - \tilde{I}_{\text{cr}})(1+M)}, \tag{18}$$

and its associated wavelength is:

$$\lambda_m = \frac{2\pi R}{k_m} = \frac{\pi h}{\sqrt{\frac{\epsilon^2}{12} + \mathrm{Ca}(\tilde{I} - \tilde{I}_{\text{cr}})(1+M)}}, \tag{19}$$

where $\epsilon = h/R \ll 1$ is a geometrical aspect ratio and $\mathrm{Ca} = U\mu_2/\gamma$ is the capillary number.

Supplementary Note 2: Strength of streaming current and electro-osmotic velocity

To better understand the instability mechanism, it is helpful to estimate the relative strength of streaming current and electro-osmotic velocity. To this end, we define the following two non-dimensional parameters:

$$\beta_{sc} = \frac{|\mathbf{i}_{\text{sc}}|}{|\mathbf{i}|}, \quad \beta_{eo} = \frac{|\mathbf{u}_{\text{eo}}|}{|\mathbf{u}|}. \tag{20}$$

To estimate β_{sc}, from supplementary equation (1) we have,

$$-\nabla p = \frac{\mathbf{u} + K_{\text{eo}}\nabla\phi}{K_{\text{h}}}, \tag{21}$$

which upon substitution into supplementary equation (2), and considering $\alpha = K_{\rm eo}^2/K_{\rm h}K_{\rm e} \ll 1$, yields:

$$\mathbf{i} = K_{\rm eo}\frac{\mathbf{u} + K_{\rm eo}\nabla\phi}{K_{\rm h}} - K_{\rm e}\nabla\phi = \frac{K_{\rm eo}}{K_{\rm h}}\mathbf{u} - K_{\rm e}(1-\alpha)\nabla\phi \approx \frac{K_{\rm eo}}{K_{\rm h}}\mathbf{u} - K_{\rm e}\nabla\phi. \tag{22}$$

Comparing the supplementary equation (22) with supplementary equation (2), yields:

$$\beta_{\rm sc} \approx \frac{K_{\rm eo}}{K_{\rm h}}\frac{Q}{I} \approx 3.17 \times 10^{-10}, \tag{23}$$

for a typical experiment ($I = 2$ mA, $Q = 200$ uL/min, water phase). Therefore the contribution of streaming current is negligible compared to Ohmic current in our experiments. A similar calculation for $\beta_{\rm eo}$ yields:

$$\beta_{\rm eo} \approx \frac{K_{\rm eo}}{K_{\rm e}}\frac{I}{Q} \approx 0.95, \tag{24}$$

under similar experimental conditions, suggesting that electro-osmotic velocity is comparable to the overall velocity. Furthermore, note that by definition $\beta_{\rm sc}\beta_{\rm eo} = \alpha \ll 1$, so that only of the effects, i.e. either streaming current or electro-osmotic velocity, could be relatively large but not both at the same time.

Finally we present an interpretation of the control parameter $\tilde{I}$ (cf. supplementary equation (16)). In our experiments, both liquids have the same conductivity. Furthermore, the water phase is both more polar and has a higher surface potential, i.e. $|\varepsilon_{\rm w}\zeta_{\rm w}| \gg |\varepsilon_{\rm o}\zeta_{\rm o}|$. Therefore,

$$|\tilde{I}| \approx \frac{\beta_{\rm eo}}{1+M} \propto \frac{U_{\rm eo}}{U}, \tag{25}$$

illustrating that the control parameter is directly proportional to the relative strength of electro-osmotic velocity in the water phase.

Supplementary Note 3: Reactions in the electrochemical cell

Potential reactions that can occur in the system are shown in **Supplementary Figure 12**. The products of any chemical reactions in the experimental cell can change the composition of the fluids. To examine how significant this effect is, we conduct a simple calculation below, which shows the amount of chemical reaction products is negligible compared to the amount of fluids. In a typical experiment ($Q = 200$ uL min^{-1}), the highest current is $I = 10$ mA and the experiment lasts for two minutes, therefore:

- The delivered charge is 10 mA $\times$ 120 s = 1.2 C

- Number of moles of dye in the cell and the cone is
  $(2\ \text{min} \times 200\ \text{uL}\,\text{min}^{-1} + 1\ \text{mL}\ ) \times 1\ \text{mg}\,\text{mL}^{-1}\ /\ (408.5\ \text{g}\,\text{mol}^{-1}) = 3.42\ \text{umol}.$
- Amount of charge that the dye can provide (5 electron reduction)
  $3.42\ \text{umol} \times 96485\ \text{C}\,\text{mol}^{-1} \times 5 = 1.65\ \text{C}$
- Number of moles of octanol in the cell and the cone:
  $(2\ \text{min} \times 200\ \text{uL}\,\text{min}^{-1} + 1\ \text{mL}\ ) \times 0.83\ \text{g}\,\text{mL}^{-1}\ /\ (130.2\ \text{g}\,\text{mol}^{-1}) = 8.9\ \text{mmol}$
- Amount of charge that the octanol can provide (1 electron reduction)
  $8.9\ \text{mmol} \times 96485\ \text{C}\,\text{mol}^{-1} = 858.7\ \text{C}$

As indicated above, the delivered charge is comparable to the charge the dye can provide, which explains the substantial color change of the dye at large current. However, the delivered charge is three orders lower than what can be provided by octanol, which suggests the composition change of octanol due to oxidation/reduction is negligible.

To further demonstrate this, we also calculate the Damkohler number to compare reaction rate and mass transfer rate. For dye, $c = 1\ \text{mg}\,\text{ml}^{-1}/(408.5\ \text{g}\,\text{mol}^{-1}) = 2.4\ \text{mM}$, we have:

$$\text{Da}_\text{I} = \frac{\text{reaction rate}}{\text{convection rate}} = \frac{\frac{I}{nFRh}}{\frac{Qc}{Rh}} = \frac{\frac{10\ \text{mA}}{1\times 96485\ \text{C}\,\text{mol}^{-1}}}{200\ \text{uL}\,\text{min}^{-1} \times 2.4\ \text{mM}} \approx 12.7 \tag{26}$$

$$\text{Da}_\text{II} = \frac{\text{reaction rate}}{\text{diffusion rate}} = \frac{\frac{I}{nFRh}}{D\frac{c}{R}} = \frac{\frac{10\ \text{mA}}{1\times 96485\ \text{C}\,\text{mol}^{-1}\times 200\ \text{um}}}{10^{-10}\ \text{m}^2\,\text{s}^{-1} \times 2.4\ \text{mM}} \approx 2.1 \times 10^6 \tag{27}$$

For octanol, $c = 0.83\ \text{g}\,\text{ml}^{-1}/(130.2\ \text{g}\,\text{mol}^{-1}) = 6.37\ \text{M}$, we have:

$$\text{Da}_\text{I} = \frac{\text{reaction rate}}{\text{convection rate}} = \frac{\frac{I}{nFRh}}{\frac{Qc}{Rh}} = \frac{\frac{10\ \text{mA}}{1\times 96485\ \text{C}\,\text{mol}^{-1}}}{200\ \text{uL}\,\text{min}^{-1} \times 6.37\ \text{M}} \approx 4.9 \times 10^{-3} \tag{28}$$

$$\text{Da}_\text{II} = \frac{\text{reaction rate}}{\text{diffusion rate}} = \frac{\frac{I}{nFRh}}{D\frac{c}{R}} = \frac{\frac{10\ \text{mA}}{1\times 96485\ \text{C}\,\text{mol}^{-1}\times 200\ \text{um}}}{10^{-10}\ \text{m}^2\,\text{s}^{-1} \times 6.37\ \text{M}} \approx 8.1 \times 10^2 \tag{29}$$

The first Damkohler number, Da_I, suggests the amount of reacted octanol can be neglected, but not for the dye. This conclusion is consistent with the above calculation. The second Damkohler number, Da_II, suggest for both octanol and dye, diffusion is much slower than reaction, indicating the reaction products can not perturb the moving interface.

Since TBA^+ and Cl^- can diffuse into from oil into water phase, to evaluate how strong this effect is, we calculate Peclet number below

$$\text{Pe} = \frac{\text{convection rate}}{\text{diffusion rate}} = \frac{RU}{D} = \frac{\frac{Q}{2\pi h}}{D} = \frac{200\ \text{uL}\,\text{min}^{-1}}{2\pi \times 200\ \text{um} \times 10^{-10}\ \text{m}^2\,\text{s}^{-1}} \approx 2.7 \times 10^4, \tag{30}$$

suggesting that the diffusion of ions across the interface may be safely ignored.

SUPPLEMENTARY REFERENCES

[1] M. Mirzadeh and M. Z. Bazant, "Electrokinetic control of viscous fingering," Phys. Rev. Lett. **119**, 174501 (2017).

[2] P. Peters, R. Van Roij, M. Z. Bazant, and P. Biesheuvel, "Analysis of electrolyte transport through charged nanopores," Phys. Rev. E **93**, 053108 (2016).

[3] L. Paterson, "Radial fingering in a hele shaw cell," J. Fluid Mech. **113**, 513–529 (1981).

[4] G. M. Homsy, "Viscous fingering in porous media," Annu. Rev. Fluid Mech. **19**, 271–311 (1987).
