# Supplementary material for: Active control of viscous fingering using electric fields
Source: Nat Commun. 2019 Sep 5;10:4002. doi: 10.1038/s41467-019-11939-7 (PMC6728344; doi:10.1038/s41467-019-11939-7)
Supplement: Supplementary file 3 — Description of Additional Supplementary Files [file 41467_2019_11939_MOESM3_ESM.pdf]

Description of Additional Supplementary Files

Supplementary movie 1. Representative examples of electrokinetic destabilization and stabilization. a). Destabilization of a hydrodynamically stable displacement. b) Stabilization of a hydrodynamically unstable displacement.

Supplementary movie 2. The patterns of the interfaces at different flow rates and current for M=1.98.
