# Supplementary material for: Active control of viscous fingering using electric fields
Source: Nat Commun. 2019 Sep 5;10:4002. doi: 10.1038/s41467-019-11939-7 (PMC6728344; doi:10.1038/s41467-019-11939-7)
Supplement: Supplementary file 2 — peer review file [file 41467_2019_11939_MOESM2_ESM.pdf]

Reviewer #1 (Remarks to the Author):

The authors demonstrate the possibility to control (promote or suppress) the fingering instability at the interface of two immiscible fluids in a Hele-Shaw cell. They achieve this control harnessing electro-osmotic flows that are generated through externally applied electric fields.

The paper is mostly experimental, while guided by clever theoretical arguments. The manuscript is very clear and the results are quite exciting.

Viscous fingering is a canonical problem in fluid mechanics, which has implication in a broad range of phenomena, including technological applications. Depending on the case, it would be advantageous to enhance or else suppress the instability. This study provides a way to do achieve such control for a fixed fluid viscosity ratio and a given geometry, a new result as far as I can judge, which should be of interest to a the readership of Nature Communications.

The paper is well written. The authors first recast classical theories and then demonstrate experimentally that they apply to their case. The figures are convincing and the agreement between theory and experiments is very good, especially knowing that the zeta potential is unknown. It is remarkable that such complex phenomena can be rationalized by an overall simple physical picture. I was very enthusiastic when I read the paper for the first time, and remains enthusiastic now that I took some time to think about its content. I would strongly recommend that the manuscript be published after the authors have considered my few queries.

While it is fine to refer the supplementary information, I find it disruptive that one must refer to Figure S3 and S4 to understand the text. Would it be possible to promote those figures, or at least part of their content to the main text?

More fundamentally, is it obvious that the roughness should reach a peak value, thereby indicating that the instability saturates in the nonlinear regime?

Similarly, do the authors have some ideas of the 3D structure of the flow. Clearly the depth average formalism does wonders, but there must be some 3D effects, given the shape of the flow profiles shown in Fig1C.

Is there an outlet in the cell?

Reviewer #2 (Remarks to the Author):

The authors report the first experimental evidence of electrokinetic control of viscous fingering instability in a thin gap (i.e., Hele-Shaw cell) during an immiscible fluid-fluid displacement. By applying an electric current, I, they manipulate the electroosmotic flows in the two phases of the fluids through the direction of I and electro-osmotic mobilities. For instance, they experimentally showed stabilization of a hydrodynamic unstable fluid displacement with a viscosity contrast, $M = \frac{\mu_1}{\mu_2} < 1$ via a positive I, while destabilization of a stable hydrodynamic situation of M > 1 using a negative current. Therefore, depending on the applied electrical current, the resultant electroosmosis flows can effectively increase or decrease the flow resistance of the defending fluid, thereby providing a useful means to control the viscous fingering instability. They also compared the experimental results of critical electric current, I_{cr}, applied with that obtained through a linear stability analysis showing the magnitude of I_{cr} is proportional to the flow rate, Q.

The topic is of great interest due to various important applications relying on effective control of viscous fingering. However, the writing style is not suitable for a general audience of different scientific background. Moreover, the experimental data under a large magnitude of dimensionless current ($\tilt{I} > 1$) surprisingly show a completely different trend of viscous fingering stability, in strong disagreement with the current theoretical linear stability analysis. While, the discussion about this discrepancy is rather limited and short (on Page 6). Also, what are the experimental results of $|\tilt{I} > 1.5 |? Do they continue to show a reverse of fingering stability? Therefore, I recommend a revision of the current manuscript to address the above points.

Reviewer #3 (Remarks to the Author):

This paper seeks to demonstrate an active method for suppressing and enhancing viscous fingering using electro-osmotic flows generated along the boundaries of the Hele-Shaw cell when applying an external electric field. A linear stability analysis summarized in the supplementary material shows that the electro-osmotic flow either adds to or opposes the pressure-driven flow thus yielding an additional pre-factor to the k term in the dispersion relation.

Experiments are then performed to confirm this theoretical prediction. The results are shown in terms of a threshold for instability in terms of current and flow rate applied and the experimental results are quite convincing for intermediate flow rates, less so for low and high flow rates, and there are also a range of outliers in the data, suggesting that the picture is more complex than that the authors are trying to convey. Although the findings are novel to my knowledge and interesting, I kept on waiting for more in-depth insight into the instability generated by the presence of the electric field and how it alters the classical viscous fingering instability. Instead the narrative was

limited to an effort to “match” experiment and theory in a fairly superficial manner. Hence I have a number of questions and comments:

1) The term electrokinetic thinning/thickening seems incorrect and confusing given that viscosity has a precise constitutive definition at the mesoscopic level. I cannot see why the fluids should thicken. Another terminology that pervades the paper is roughness. Given the considerable sensitivity of viscous fingering to roughness of the boundaries, I find this terminology rather misleading: roughness here is meant to describe the amplitude of the non-axisymmetric interfacial perturbation. Why not be precise, particularly as roughness suggests stochasticity which I don’t think enters the problem.

2) There are a number of vague statements in the paper regarding interfaces being more or less unstable, but there is generally a lack of precision as to what this means. Note that in the radial systems, I think mode 1 is neutrally stable and stabilisation starts at mode 2. Now, the dispersion relation presented does not look like the full the radial analysis, see e.g. Paterson (1981). More unstable here seems to refer to a larger dominant unstable wavenumber. This is fine in the context of the mechanism described as long as surface tension is constant but with the rather complex electrochemistry going on, I would expect surface tension to vary with the imposed current, in which case the mechanism leading to an increased band of unstable modes could be due to a reduction in surface tension. There is a vast literature on polarization effects of the interface between two immiscible electrolyte solutions (ITIES) and I was surprised that this was not considered (see, e.g. reviews by H.H. Girault).

3) The primary motivation is to enhance/suppress fingering instabilities but the system studied here only seems to produce fingers of very small amplitude and thus its interfacial instabilities cannot be particularly detrimental to applications. Now, even with the largest currents it does not appear possible to drive highly indented patterns.

4) Looking at the experimental images, I wonder about three-dimensional effects (particularly with the large wavenumbers in some of the experiments. It would be helpful to add scales on representative experimental images. The two fluids have different wettabilities so there is likely films forming during the displacement process. The colour intensity appears to vary in various results (SI, e.g. Fig. S5) and this may suggest different film thicknesses left behind on the plates, although the origin of these variations may originate elsewhere. If I understand correctly, the entire theoretical picture is based on the total displacement of one fluid by another. How is this influenced by the presence of films? Does it still make sense as the EDL driving the flows would have a completely different structure?

5) I would have been more convinced by the comparison between experiments and linear stability analysis if the most unstable wave number had been systematically compared to the dominant mode in the experiments (in viscous fingering, the most unstable mode tends to be set by linear stability analysis, even for nonlinear growth, however, it usually varies with radial position except in Zheng et al. where the top plate is lifted.) A more careful comparison seems essential particularly in view of the considerable number of outlying results that do not fit the simple theoretical picture presented.

6) Finally, a number of the patterns shown are not directly reminiscent of classic viscous fingering suggesting that the application of the electric field in this rather complex system acts on the instability (always shown in the nonlinear growth regime) in a more complex manner than suggested by the linear stability analysis

Response to Reviewers' Comments: Active Control of Viscous Fingering Using Electric Fields

Tao Gao,[1,*] Mohammad Mirzadeh,[1,*] Peng Bai,[1,†]
Kameron M. Conforti,[1] and Martin Z. Bazant[1,2,‡]

[1]*Department of Chemical Engineering, Massachusetts Institute of Technology, MA 02139.*

[2]*Department of Mathematics, Massachusetts Institute of Technology, MA 02139.*

REVIEWER 1

The authors demonstrate the possibility to control (promote or suppress) the fingering instability at the interface of two immiscible fluids in a Hele-Shaw cell. They achieve this control harnessing electro-osmotic flows that are generated through externally applied electric fields. The paper is mostly experimental, while guided by clever theoretical arguments. The manuscript is very clear and the results are quite exciting.

Viscous fingering is a canonical problem in fluid mechanics, which has implication in a broad range of phenomena, including technological applications. Depending on the case, it would be advantageous to enhance or else suppress the instability. This study provides a way to do achieve such control for a fixed fluid viscosity ratio and a given geometry, a new result as far as I can judge, which should be of interest to a the readership of Nature Communications.

The paper is well written. The authors first recast classical theories and then demonstrate experimentally that they apply to their case. The figures are convincing and the agreement between theory and experiments is very good, especially knowing that the zeta potential is unknown. It is remarkable that such complex phenomena can be rationalized by an overall simple physical picture. I was very enthusiastic when I read the paper for the first time, and remains enthusiastic now that I took some time to think about its content. I would strongly recommend that the manuscript be published after the authors have considered my few queries.

Reply: We thank the reviewer for the kind comments, and are grateful for his/her appreciation of the significance and novelty of our work.

Q1: While it is fine to refer the supplementary information, I find it disruptive that one must refer to Figure S3 and S4 to understand the text. Would it be possible to promote those figures, or at

least part of their content to the main text?

Reply: We thank the reviewer for the suggestion. We did not incorporate the temporal evolution pictures of the interface because there are too many figures. In the revised manuscript, we include the pictures at several representative times to keep the manuscript concise and make it easier for readers to understand the texts, while keeping most of the results in the supporting materials to make the story complete.

Q2: More fundamentally, is it obvious that the roughness should reach a peak value, thereby indicating that the instability saturates in the nonlinear regime?

Reply: In our experiments, we observed that roughness reaches a peak value when the interface motion is unstable (e.g. see Fig. S9). This is not an obvious point, but we have not explored the exact cause of this observation. Indeed the linear stability theory predicts an exponential growth of individual modes and, by extension, the roughness measure. In the nonlinear regime, the growth is slowed but is expected to continue due to recurrent tip-splitting events in radial geometry [1]. More recently, studying of viscous fingering with miscible liquids, have revealed interesting patterns [2]. In these experiments, the instability grows in proportion to a region of stable growth behind the fingers. The ratio between these two regions depends on the viscosity contrast (see figures 2 and 3 in [2]). For such a "proportional growth" mechanism, our definition of roughness is expected to asymptotically reach a constant value. It is conceivable that similar phenomena might be at work in our experiments. This is certainly an interesting question in itself but we have not pursued the exact cause in this work. Instead, our primary goal has been identifying the role of electrokinetics in controlling viscous fingering.

Q3: Similarly, do the authors have some ideas of the 3D structure of the flow. Clearly the depth average formalism does wonders, but there must be some 3D effects, given the shape of the flow profiles shown in Fig1C

Reply: It is plausible to expect 3D flow structures close to the wall, especially when the hydraulic and electro-osmotic flows are in opposite directions. In our experiments, the Debye length ($\lambda_D = \sqrt{\varepsilon k_B T / \sum_i c_i z_i^2 e^2} \approx 10$ nm) is much smaller than the cell gap ($h = 200$ um),

and the electro-osmotic velocity is nearly uniform across the gap. Therefore, one might expect a flow reversal region close to the wall which could in turn affect the contact line motion. If these flow structures become dominant, they might disturb the contact line motion or promote a wetting transition. Clearly our depth-averaged analysis does not take such possibilities into account and instead assumes the contact line is moving with the total average velocity. This average velocity is well described by summing the hydraulic and electro-osmotic velocities. In our experiments, we have not observed any major indication to discard the depth averaged model. If 3D flow structures do exist, they seem to be weak enough to not significantly impact the contact line motion. Nonetheless, despite these possible shortcomings and the complicated nature of the problem, it is quite remarkable that our simple theory is still able to capture the essence of the instability mechanism. Further 3D simulations or detailed experimental measurements of concentration profile could shed more light on the 3D flow structure and its impact on the contact line motion and the instability in general.

Q4: Is there an outlet in the cell?

Reply: Yes. The Hele-Shaw cell (stacked glass plates) is contained in a shallow round container made of acrylic. During the experiment, the water coming out from the Hele-Shaw cell will enter the annulus-shaped reservoir between the inner wall of the container and the Hele-Shaw cell. After each experiment, we drain the fluid in the reservoir.

REVIEWER 2

The authors report the first experimental evidence of electrokinetic control of viscous fingering instability in a thin gap (i.e., Hele-Shaw cell) during an immiscible fluid-fluid displacement. By applying an electric current, I, they manipulate the electroosmotic flows in the two phases of the fluids through the direction of I and electro-osmotic mobilities. For instance, they experimentally showed stabilization of a hydrodynamic unstable fluid displacement with a viscosity contrast, $M = \frac{\mu_1}{\mu_2} < 1$ via a positive I, while destabilization of a stable hydrodynamic situation of $M = \frac{\mu_1}{\mu_2} > 1$ using a negative current. Therefore, depending on the applied electrical current, the resultant electroosmosis flows can effectively increase or decrease the flow resistance of the defending fluid,

thereby providing a useful means to control the viscous fingering instability. They also compared the experimental results of critical electric current, I_{cr}, applied with that obtained through a linear stability analysis showing the magnitude of I_{cr} is proportional to the flow rate, Q.

Reply: We thank the reviewer for the kind comments, and are very grateful for his understanding and appreciation of the significance and novelty of this work.

Q1: The topic is of great interest due to various important applications relying on effective control of viscous fingering. However, the writing style is not suitable for a general audience of different scientific background.

Reply: We thank the reviewer for the comments. In the revised manuscript, we moved some equations to make the paper less technical. Also, we tried to add more background knowledge and discussion in the texts to make the results more understandable. In addition, we add more discussion on the potential applications of the idea for broader audience with various background.

Q2: Moreover, the experimental data under a large magnitude of dimensionless current ($\tilde{I} > 1$) surprisingly show a completely different trend of viscous fingering stability, in strong disagreement with the current theoretical linear stability analysis. While, the discussion about this discrepancy is rather limited and short (on Page 6).

Reply: We thank the reviewer for the critical comment. In the revised manuscript, we have added additional discussion regarding the anomalous points at large currents. These anomalies cannot be explained by our linear stability theory. A possible destabilizing effect could be related to charge build-up at the interface and the resulting Maxwell stresses. These Maxwell stresses could become noticeable at relatively large electric fields, typically around $E \sim 10$ kV/cm [3–5], and are the cause of "electro-hydrodynamic (EHD) instability". However, EHD effects are fundamentally different from the EK effects we have described in this paper. First, the applied field in our experiments are considerably weaker, with the strongest value being around $E \sim 0.3$ kV/cm. Second, EHD effects would always be destabilizing [5]. This is evident in the scaling of Maxwell stress, $\tau_M \propto |E|^2$, which suggests the direction of electric field does

not affect the instability. In contrast, electric fields appear linearly in our theory (see eq. 2 in the main text). This linear relationship is paramount to controllability: electric fields are stabilizing in one direction and destabilizing in the reverse direction. In our experiments we clearly see these direction-dependent effects of electric fields, e.g. see Fig. R1, R3, and R4.

Our work is the first study that shows experimental evidence of electrokinetic control of viscous fingering. The theory based on linear stability analysis is in good agreement with the phenomena observed at low and moderate currents. Furthermore, we believe our work lays the foundation for future research. Developing a comprehensive theory that captures these non-linear effects at high voltage is certainly exciting. It is, however, beyond the scope of this work. We have added the above qualitative analysis and discussion into the revised manuscript, hoping they can provide some sense to readers, as well as shed some light on future research.

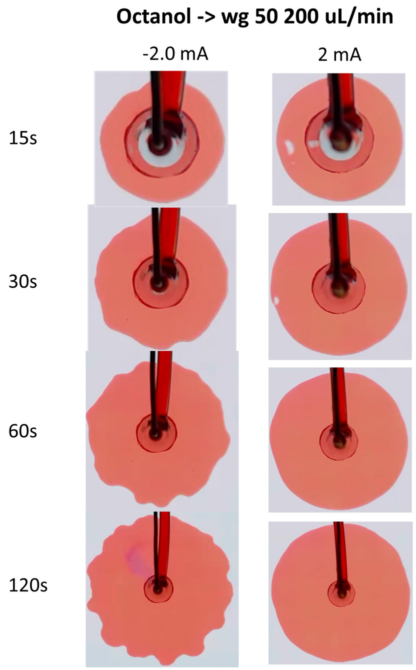

FIG. R1. Interfaces of oil pushing water-glycerol 50/50 at +2 mA and -2 mA

Q3: Also, what are the experimental results of $\tilde{I} > 1.5$. Do they continue to show a reverse of fingering stability? Therefore, I recommend a revision of the current manuscript to address the above points.

Reply: We thank the reviewer for the comment. To answer this question, we first look at the raw experimental data at different viscosity ratios. In our experiments, the maximum current that we can apply is bounded by instrument limitation ($I = 10$ mA maximum). For experiments at different viscosity ratios, we only sometimes observe the anomalous behavior within the limits of our instrument. In some experiments, the anomalous phenomena occurs at intermediate currents. In these cases, we observe similar anomalous behavior at even higher currents. For example, when applying positive current to stabilize the interface of oil pushing water (Fig. R2), we started to see anomalous behavior at $I = +4$ mA. Increasing current to $I = +6$ mA further destabilizes the interface. When applying negative current to destabilize the interface of oil pushing water-glycerol 50/50 (Fig. R3), we first observed the interface becoming unstable due to electro-kinetic effects at intermediate currents ($I = -0.5$ mA to $I = -2$ mA), indicated by the appearance of fingers from all directions. When current was increased to $I = -4.0$ mA, the system enters the anomalous regime, where most part of the interface becomes smooth, leaving only some instability in the upper-left and bottom-right direction. Further increasing the current to $I = -8.0$ mA strengthen this trend, indicated by the disappearance of fingers at all directions.

In some other experiments, we did not observe any anomalous phenomena even the current has reached instrument limit. For example, in oil pushing water-glycerol 50/50 experiments (Fig. R4), we did not observe the anomalous interface stability as observed in Fig. R2. The color change indicates the redox reaction of dye. In oil pushing water-glycerol 60/40 experiments(Fig. R5), we did not observe the anomalous interface stability as observed in Fig. R3.

In summary, the anomalous behavior is inconsistent with our theory but only occurs at intermediate and high currents. When the anomalous behavior is triggered, further increasing the magnitude of the current will strengthen the trend. Due to instrument limitation (maximum current 10 mA), we were unable to collect systematic results to better analyze the pattern and conditions of these anomalous behavior. The emergence of these anomalous points might be related, at least in part, to EHD instability discussed above. However, we have not fully investigated these anomalous behavior as they were outside the main scope of this work. Although we cannot provide a conclusive description, we have decided to report these experimental observations to encourage further research on this topic.

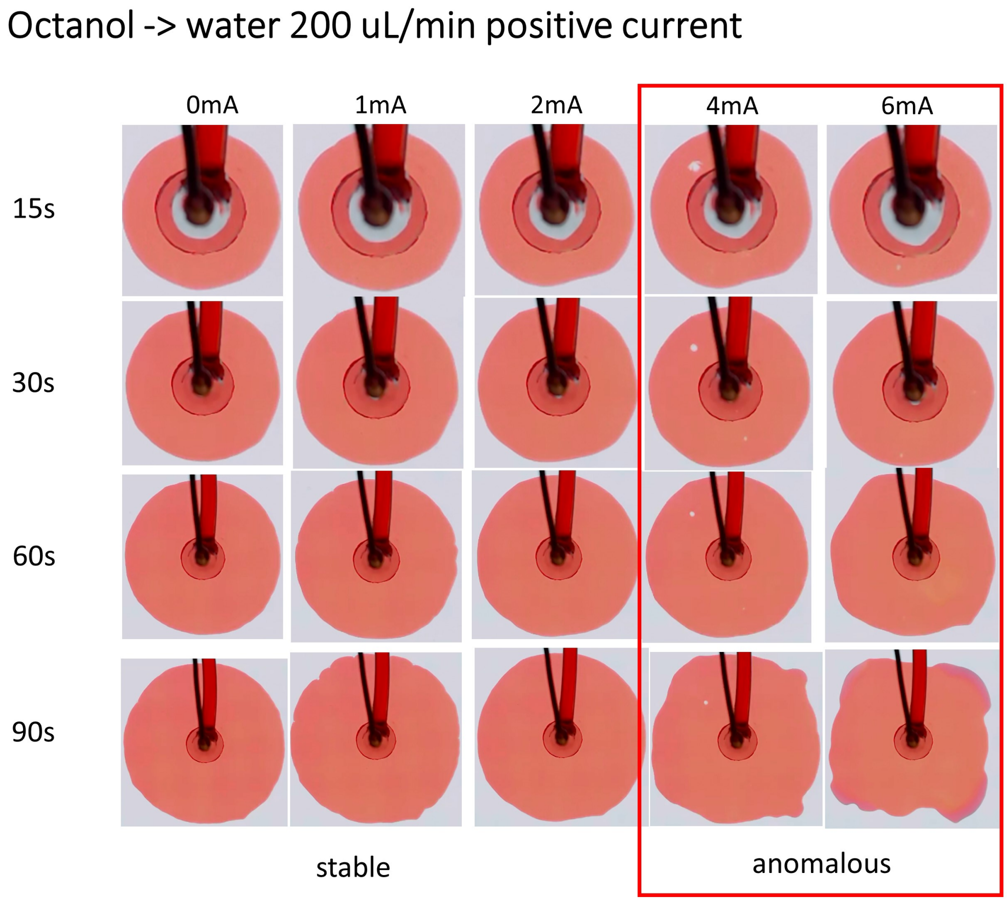

FIG. R2. Interfaces for oil pushing water at different positive currents

REVIEWER 3

This paper seeks to demonstrate an active method for suppressing and enhancing viscous fingering using electro-osmotic flows generated along the boundaries of the Hele-Shaw cell when applying an external electric field. A linear stability analysis summarized in the supplementary material shows that the electro-osmotic flow either adds to or opposes the pressure-driven flow thus yielding an additional pre-factor to the k term in the dispersion relation.

Experiments are then performed to confirm this theoretical prediction. The results are shown in terms of a threshold for instability in terms of current and flow rate applied and the experimental results are quite convincing for intermediate flow rates, less so for low and high flow rates, and there are also a range of outliers in the data, suggesting that the picture is more complex than that the authors are trying to convey. Although the findings are novel to my knowledge and interesting, I kept on waiting for more in-depth insight into the instability generated by the presence of the

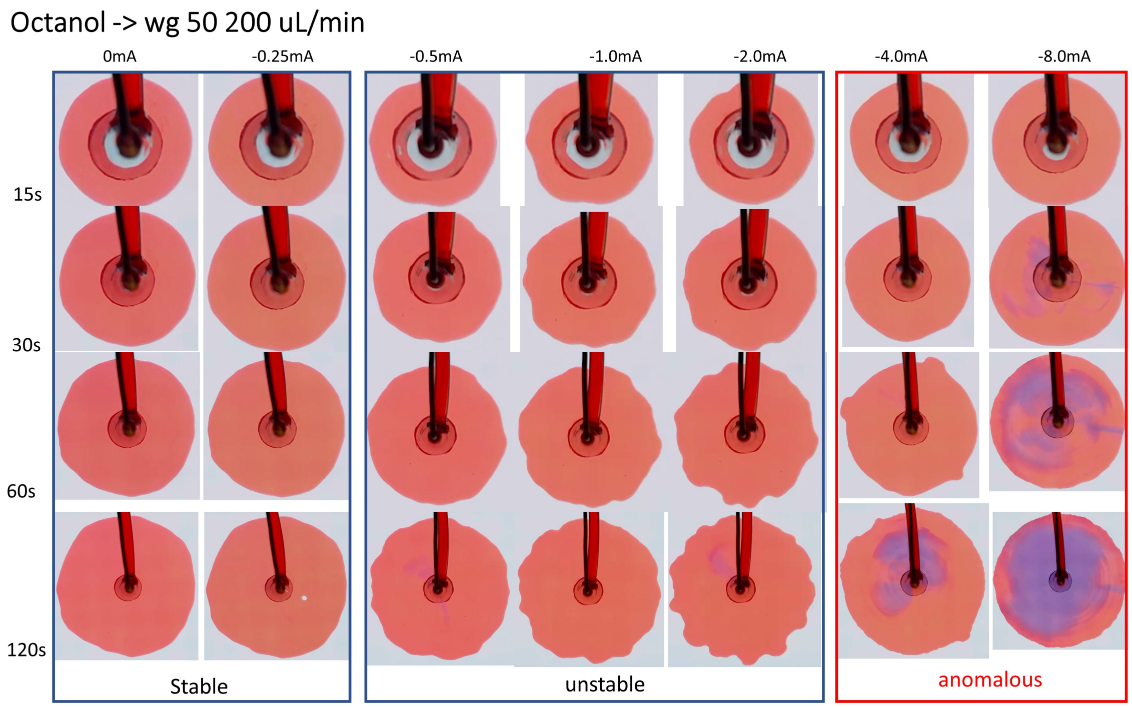

FIG. R3. Interfaces for oil pushing water-glycerol 50/50 at different negative currents

electric field and how it alters the classical viscous fingering instability. Instead the narrative was limited to an effort to match experiment and theory in a fairly superficial manner. Hence I have a number of questions and comments:

Reply: We thank the reviewer for the critical comments. We have made adjustments to the manuscript and discuss our replies below.

Q1: The term electrokinetic thinning/thickening seems incorrect and confusing given that viscosity has a precise constitutive definition at the mesoscopic level. I cannot see why the fluids should thicken. Another terminology that pervades the paper is roughness. Given the considerable sensitivity of viscous fingering to roughness of the boundaries, I find this terminology rather misleading: roughness here is meant to describe the amplitude of the non-axisymmetric interfacial perturbation. Why not be precise, particularly as roughness suggests stochasticity which I dont think enters the problem.

Reply: We agree with the reviewer on the ambiguity of our terminologies. To address these comments, we have decided to modify our terminologies to "apparent electrokinetic thin-

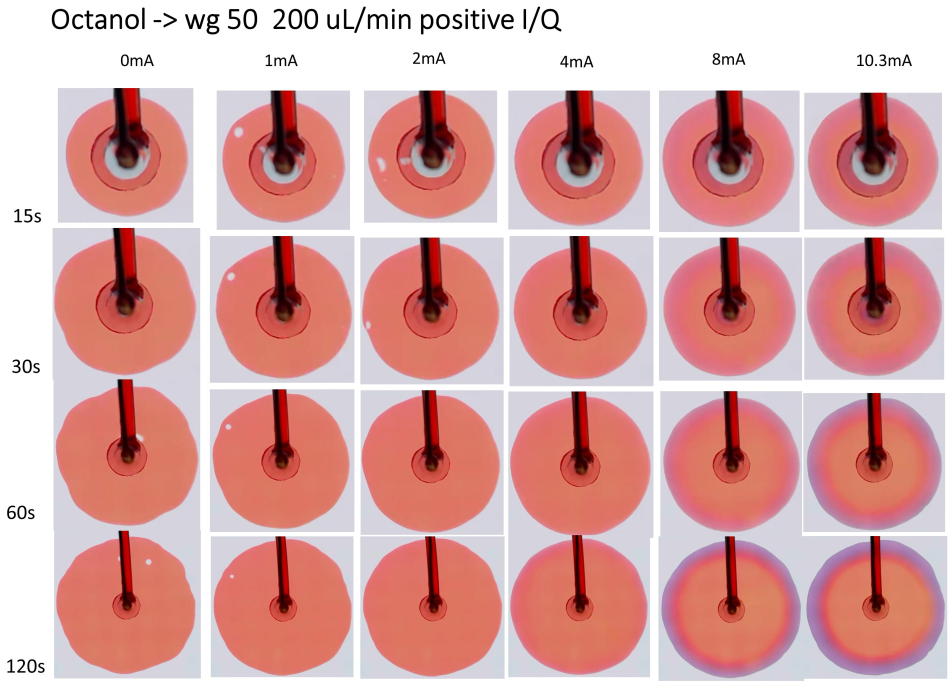

FIG. R4. Interfaces for oil pushing water-glycerol 50/50 at different positive currents

ning/thickening" and "interfacial roughness" as discussed below.

The electric field, and the associated electrokinetic phenomena, can affect the fluid viscosity in two ways, which (quite confusingly) are often referred to as "viscoelectric" and "electroviscous" effects (e.g. see chapter 5 of [6]). The viscoelectric effect refers to an actual enhancement of molecular viscosity of water due to structural rearrangement of water molecules in strong field [7–9]. This is *not* what we refer to in the manuscript. The "electroviscous" effect refers to an *apparent* enhancement of the viscosity of an electrolyte when it is forced through charged capillaries or porous media [10, 11]. This apparent enhancement is due to additional electro-osmotic drag that is generated by the self-induced streaming potential.

What we call "apparent electrokinetic thinning/thickening" is related to the change in the *apparent viscosity* due to electrokinetic coupling; much like the electroviscous effect, there is no actual change of fluid viscosity. However, the main difference is that we externally control the electric field which drives electro-osmotic flows. As a result, we can (effectively) either enhance the apparent viscosity or suppress it by choosing the relative direction of electro-osmotic and hydraulic flows.

We also agree with the reviewer's comment that the choice of "roughness" without any

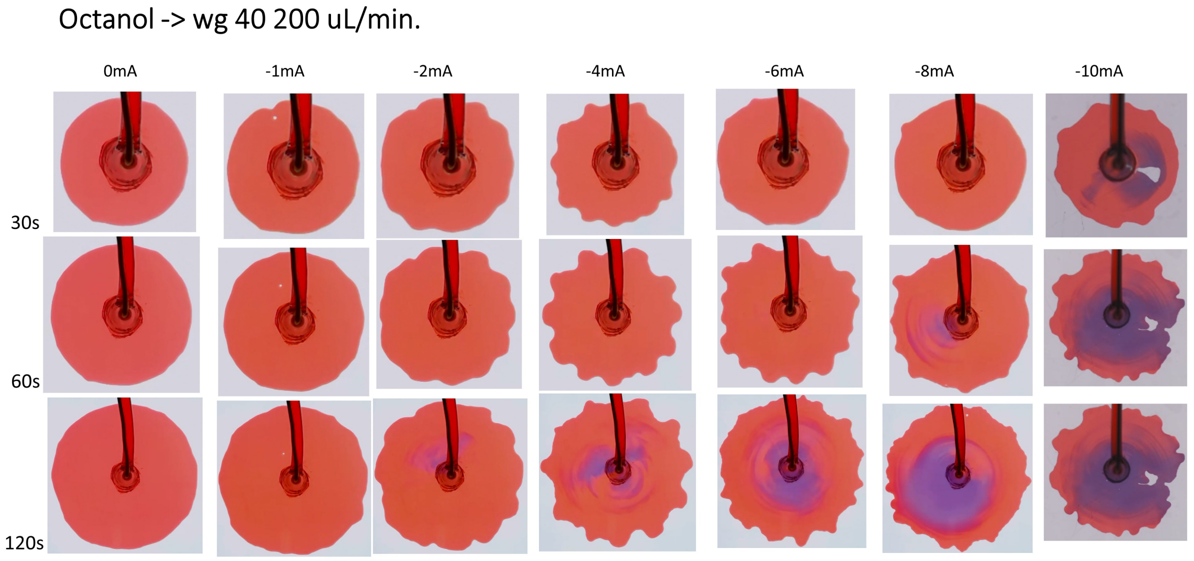

FIG. R5. Interfaces for oil pushing water-glycerol 60/40 at different negative currents

further context is confusing. What we call roughness, is not the intrinsic surface roughness, but rather the root mean square (RMS) measure of interfacial roughness. To avoid confusion, we have changed the definition to "interfacial roughness" to provide more context.

Q2: There are a number of vague statements in the paper regarding interfaces being more or less unstable, but there is generally a lack of precision as to what this means. Note that in the radial systems, I think mode 1 is neutrally stable and stabilisation starts at mode 2. Now, the dispersion relation presented does not look like the full the radial analysis, see e.g. Paterson (1981). More unstable here seems to refer to a larger dominant unstable wavenumber. This is fine in the context of the mechanism described as long as surface tension is constant but with the rather complex electrochemistry going on, I would expect surface tension to vary with the imposed current, in which case the mechanism leading to an increased band of unstable modes could be due to a reduction in surface tension. There is a vast literature on polarization effects of the interface between two immiscible electrolyte solutions (ITIES) and I was surprised that this was not considered (see, e.g. reviews by H.H. Girault).

Reply: We thank the reviewer for the critical discussion of our theory. We are also thankful for the ITIES references which have been incorporated in the main text with proper discussion. The dispersion relation in equation S5 is the result of linear stability analysis in radial geometry, originally done in our previous paper [12]. We have included the detailed derivation for completeness.

To address the remaining concerns with regards to changes in surface tension, we note the following. First, we did a thorough analysis on the influence of reaction in our experiment (see supporting information for detailed discussion). During the experiment, redox reaction needs to happen at the electrode/electrolyte to sustain the current. For the oil phase, both the dye (oil red) and the solvent (octanol) can react. In the experiment, the maximum current we applied is $I = 10$ mA. At this current, the Damkohler number, measuring the ratio of reaction rate and convection rate, is 12.7 and 4.9 $\times 10^{-3}$ for the dye and octanol, respectively. This suggests the reaction of octanol is negligible. Since the concentration of the dye is very small (2.4 mM), its reaction should also not change the bulk properties of the electrolyte. Furthermore, the dye is not a surfactant, and therefore we do not expect a change in surface tension due to interfacial adsorption.

It is however plausible that interfacial tension is reduced in our experiments due to electrocapillarity effect. For an unstable displacement, this possible reduction will in fact shift the most unstable wave number to higher values. However, such a reduction cannot explain the stabilization. Indeed electrocapillarity always predicts a reduction in surface tension (which is destabilizing), independent of the field direction. However, we clearly see different trends when the field direction is reversed (e.g. see Fig. R1).

We agree with the reviewer that our theory is an idealization of a very complex phenomena. However, we wish to clarify that the primary goal in this paper was to demonstrate the role of electrokinetics in controlling viscous fingering. Our linear stability theory predicts a linear boundary (given via equation 2 in dimensional form) between stable and unstable displacement (see Fig. 4 in the main text). Even without resorting to linear stability theory, it is possible to predict the same boundary by only considering pressure distribution ahead and behind the interface. This physical argument, which we present in the main text, is an extension of Hill's original argument [13] for classical viscous fingering. His theory also ignores surface tension and arrives at the prediction for the stability boundary that was later obtained via linear stability theory of Saffman & Taylor [14] and Chuoke *et al.* [15].

To summarize, electrocapillarity effects will likely shift the most unstable wavenumber to higher values. We have included a discussion of these effects, with references to ITIES, in the main text. However, the boundary between stable and unstable displacement, is predominantly dictated by pressure distribution ahead and behind the interface. When electrokinetic effects are present, "apparent electrokinetic thinning/thickening" affects this pressure distribution and therefore shifts the boundary.

Q3: The primary motivation is to enhance/suppress fingering instabilities but the system studied here only seems to produce fingers of very small amplitude and thus its interfacial instabilities cannot be particularly detrimental to applications. Now, even with the largest currents it does not appear possible to drive highly indented patterns.

Reply: We thank the reviewer for the comment. This work is intended to be a fundamental study to demonstrate the new possibility to control viscous fingering with given geometry and fluids. The experimental apparatus, selection of electrolytes and conditions are all chosen to serve this purpose. With systematic experiments, we clearly demonstrate the suppression of instability at positive currents and enhancement of instability at negative currents. Quantitative analysis of the geometrical shape of the interface confirms the suppression/enhancement of interface when external electric field is applied. These results open a new window for tuning viscous fingering for different applications. To demonstrate the feasibility of the idea for practical applications, optimization of the apparatus, electrolytes and conditions are indispensable to achieve the desired outcome. This is outside of the scope of the current work.

Q4: Looking at the experimental images, I wonder about three-dimensional effects (particularly with the large wavenumbers in some of the experiments. It would be helpful to add scales on representative experimental images. The two fluids have different wettabilities so there is likely films forming during the displacement process. The colour intensity appears to vary in various results (SI, e.g. Fig. S5) and this may suggest different film thicknesses left behind on the plates, although the origin of these variations may originate elsewhere. If I understand correctly, the entire theoretical picture is based on the total displacement of one fluid by another. How is this influenced by the presence of films? Does it still make sense as the EDL driving the flows would have a completely different structure?

Reply: We thank the reviewer for the comments. The slight difference in color is most likely due to the interference of ambient light because the experiments were done in different dates and time. That being said, we expect some three dimensional effects to be present when the electro-osmotic and hydraulic flows are in opposite directions. This is due to possible flow reversal close to the wall caused by the locally stronger electro-osmotic flow (see Fig. 1 in the main text). Such three dimensional effects might affect the contact-line motion.

However, in our experiments we have not observed any anomalous motion that might be attributed to three-dimensional flows. Nevertheless, we agree with the reviewer that this is an important point to note and have added a paragraph that includes a discussion of possible 3D effects. A more detailed analysis of the problem would require either 3D simulations or depth-resolved experiments, both of which were outside the scope of this paper.

Wetting transition could lead to formation of thin films above a critical capillary number, typically in the range of $\mathrm{Ca}^{\star} \sim 10^{-2} - 10^{-1}$, depending on the viscosity ratio and static contact angle [16–19]. In our system, we expect the this critical capillary number to be around $\mathrm{Ca} \sim 10^{-2}$ (e.g. see fig 12 of [17]). We have not considered the existence of such thin films in our theory or interpretation of experimental data since the capillary number in our experiments is typically much smaller than this critical value, $\mathrm{Ca} \sim 10^{-5}$ (see supporting information). Even in most the extreme case (highest flow rate and at the inlet condition), the capillary number is roughly $\mathrm{Ca} \sim 5 \times 10^{-4}$, which is still 1-2 orders of magnitude smaller than the critical value.

It is therefore safe to assume that thin wetting films are likely not present in our experiments. Even if they do, it might still be possible to use the present theory with a modification. IN a modified theory, the zeta potential in the oil phase is understood as an "effective" value that also includes contribution from the interfacial zeta potential. However, we like to point that the experimental data seems rather insensitive to the exact value of oil zeta potential due to the relatively small dielectric constant of the oil phase (see fig. S10b). Nevertheless, we believe this is a valid concern and have included a brief discussion of thin wetting films and their possible impact on interpreting the results in the main text.

Q5: I would have been more convinced by the comparison between experiments and linear stability analysis if the most unstable wave number had been systematically compared to the dominant mode in the experiments (in viscous fingering, the most unstable mode tends to be set by linear stability analysis, even for nonlinear growth, however, it usually varies with radial position except in Zheng

et al. where the top plate is lifted.) A more careful comparison seems essential particularly in view of the considerable number of outlying results that do not fit the simple theoretical picture presented.

Reply: We thank the reviewer for the comments. We agree with the reviewer that comparing the dominant mode in the experiment with the most unstable wave number predicted by the theory would be the best way to validate the theory. We actually did some analysis. For example, Fig. R6 gives the Fourier transform of a symmetric interface obtained in the experiment. The dominant mode can be easily identified based on the magnitudes of the modes. For this particular case, it is mode $k = 11$. However, for most experiments, the interface is not so symmetric (Fig. S1, S3 and S4), which makes the same analysis difficult. For example, Fig. R7 and Fig. R8 give the Fourier transform of interfaces of oil pushing water-glycerol 60/40 at $I = -3$ mA and $I = -6$ mA, respectively. As can be seen, it is not obvious which mode is dominating.

In general, such a modal analysis is easily contaminated by any sufficiently large initial perturbation. The linear stability theory, and the resulting dispersion relations are strictly valid for an initially circular interface. Even in the context of classical viscous fingering, such an analysis is rarely tried. For these reasons, we have decided to only check the stability boundary given by equation 2 in the main text. This equation could also be derived from a more general argument based on pressure distribution, similar to Hill's original argument [13], and is therefore less susceptible to experimental errors and uncertainties.

Q6: Finally, a number of the patterns shown are not directly reminiscent of classic viscous fingering suggesting that the application of the electric field in this rather complex system acts on the instability (always shown in the nonlinear growth regime) in a more complex manner than suggested by the linear stability analysis

Reply: We thank the reviewer for the comments. As discussed earlier, our primary goal in this paper is to establish the role of electro-osmotic flows on the stability of the viscous fingering. We also agree with the reviewer that, more generally, the effect of electric field could be nonlinear, e.g. if we also include electrocapillarity or EHD effects through Maxwell stresses. Maxwell stresses become dominant at higher field strength which might be relevant to some

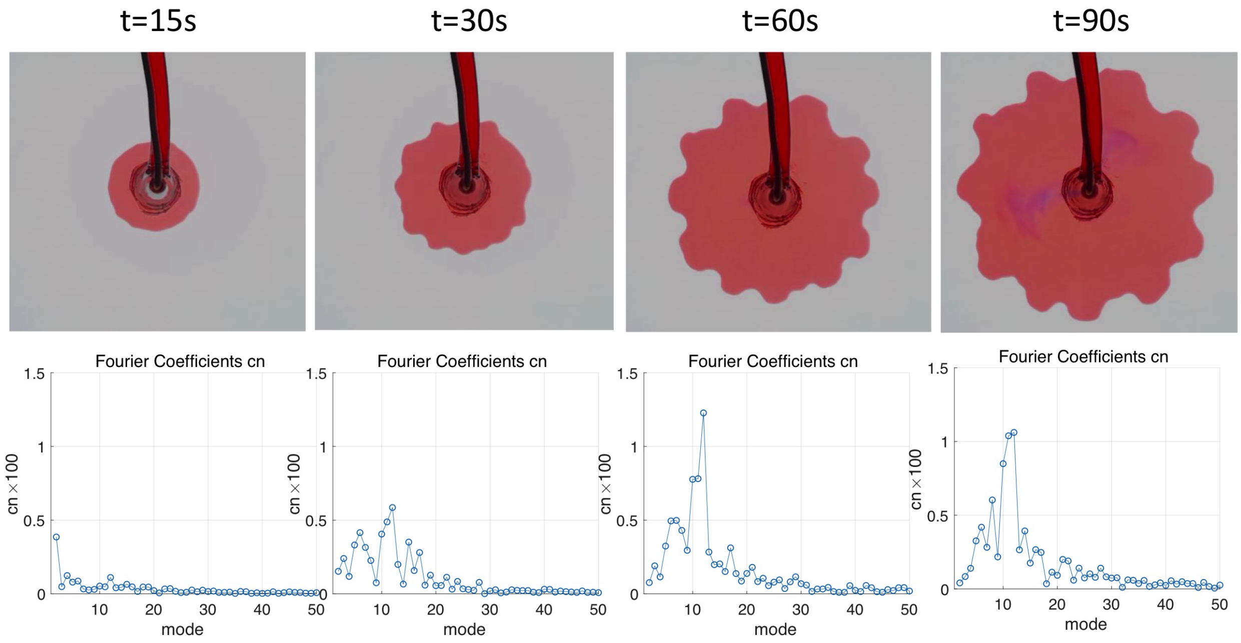

FIG. R6. Fourier Transform of interface of oil push water-glycerol 60/40 at -4 mA

of our anomalous results. However, both electrocapillrity and EHD effects scale quadratically with the electric field and therefore cannot explain the stable/unstable transition as the field direction is reversed, e.g. see Fig. R1. We have added a discussion of these shortcomings in the main text. We believe our work provides only a first look into a complex, albeit exciting phenomena and we hope these discussions further encourage other researchers to test and try some of these ideas.

---

* T.G. and M.M. contributed equally to this work.
† Current address: Department of Energy, Environmental, and Chemical Engineering, Washington University in St. Louis, MO 63130.
‡ Corresponding author: bazant@mit.edu

[1] L. Paterson, Journal of Fluid Mechanics **113**, 513 (1981).
[2] I. Bischofberger, R. Ramachandran, and S. R. Nagel, Nature communications **5**, 5265 (2014).
[3] G. Taylor and A. McEwan, Journal of Fluid Mechanics **22**, 1 (1965).
[4] J. Melcher and G. Taylor, Annual review of fluid mechanics **1**, 111 (1969).

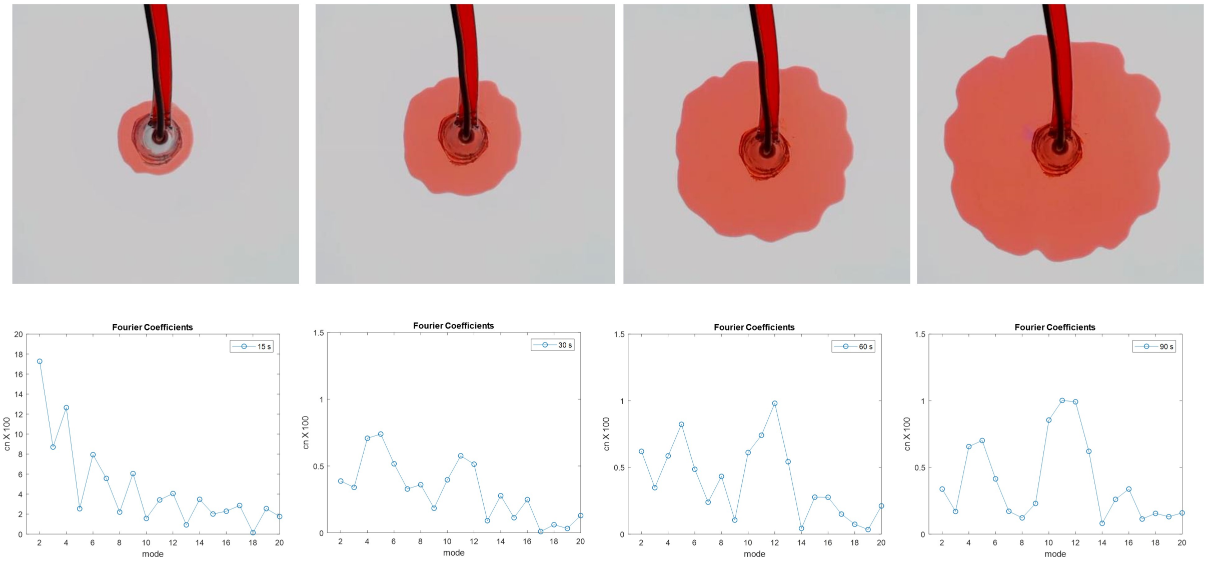

FIG. R7. Fourier Transform of interface of oil push water-glycerol 60/40 at -3 mA

[5] J. R. Melcher, *Field-coupled surface waves* (MIT, 1963).

[6] R. J. Hunter, *Zeta potential in colloid science: principles and applications*, Vol. 2 (Academic press, 2013).

[7] J. Lyklema and J. T. G. Overbeek, Journal of Colloid Science **16**, 501 (1961).

[8] J. N. Israelachvili, Journal of colloid and interface science **110**, 263 (1986).

[9] W.-L. Hsu, D. J. Harvie, M. R. Davidson, D. E. Dunstan, J. Hwang, and H. Daiguji, The Journal of Physical Chemistry C **121**, 20517 (2017).

[10] S. Levine, J. Marriott, G. Neale, and N. Epstein, Journal of Colloid and Interface Science **52**, 136 (1975).

[11] W. R. Bowen and F. Jenner, Journal of colloid and interface science **173**, 388 (1995).

[12] M. Mirzadeh and M. Z. Bazant, Physical review letters **119**, 174501 (2017).

[13] S. Hill *et al.*, Chemical Engineering Science **1**, 247 (1952).

[14] P. G. Saffman and G. I. Taylor, Proc. R. Soc. Lond. A **245**, 312 (1958).

[15] R. L. Chuoke, P. van Meurs, and C. van der Poel, Pet. Trans. AIME **216**, 188 (1959).

[16] R. L. Hoffman, Journal of colloid and interface science **50**, 228 (1975).

[17] M. Fermigier and P. Jenffer, Journal of colloid and interface science **146**, 226 (1991).

[18] D. Bonn, J. Eggers, J. Indekeu, J. Meunier, and E. Rolley, Reviews of modern physics **81**, 739 (2009).

[19] R. Ledesma-Aguilar, A. Hernández-Machado, and I. Pagonabarraga, Physical review letters **110**, 264502 (2013).

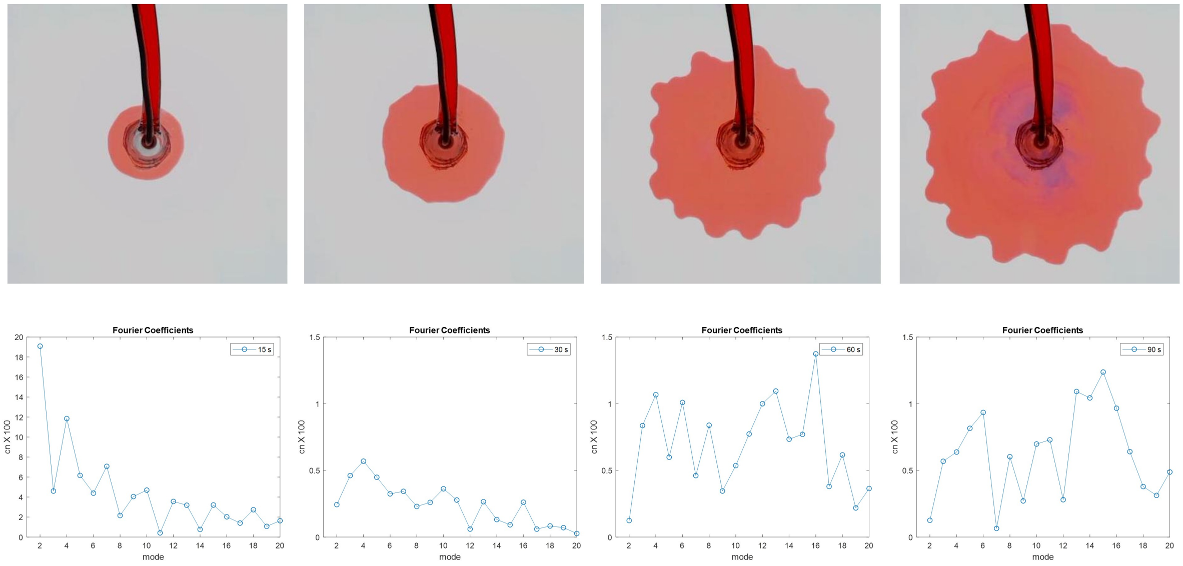

FIG. R8. Fourier Transform of interface of oil push water-glycerol 60/40 at -6 mA

Reviewer #1 (Remarks to the Author):

I am satisfied by the response of the authors. I am happy to recommend the publication of this manuscript.

Reviewer #3 (Remarks to the Author):

The authors have carefully considered my comments and revised their paper to add additional information as required. I am happy to recommend publication.

I have a few remarks about the response to referee 1 about 'proportionate growth'. This term is rather descriptive and does not reflect the dynamical origin of this effect, which is not new. Essentially, my understanding is that proportionate growth in a radial system is an invariant state of the dynamical system (i.e. reached after initial transients) which can be likened to a steady state in a linear geometry (where the velocity of the interface is constant rather than decreasing as in the radial geometry). In radial geometries, it consists of self-similar patterns of fingers and is rather rare because in these systems, the nonlinear interface growth is typically disordered. Such states were calculated theoretically for viscous fingering back in the early 1990s by Ben Amar (albeit not their stability) and observed thereafter in experiments in sector geometries (Lajeunesse & Couder). For more details on this point, see the recent paper by Vaquero-Stainer et al Self-similar and disordered front propagation in a radial Hele-Shaw channel with time-varying cell depth Phys. Rev. Fluids 4, 064002.

A. Juel

REVIEWERS' COMMENTS:

Reviewer #1 (Remarks to the Author):

I am satisfied by the response of the authors. I am happy to recommend the publication of this manuscript.

Reviewer #3 (Remarks to the Author):

The authors have carefully considered my comments and revised their paper to add additional information as required. I am happy to recommend publication.

Reply: Thank you!

I have a few remarks about the response to referee 1 about 'proportionate growth'. This term is rather descriptive and does not reflect the dynamical origin of this effect, which is not new. Essentially, my understanding is that proportionate growth in a radial system is an invariant state of the dynamical system (i.e. reached after initial transients) which can be likened to a steady state in a linear geometry (where the velocity of the interface is constant rather than decreasing as in the radial geometry). In radial geometries, it consists of self-similar patterns of fingers and is rather rare because in these systems, the nonlinear interface growth is typically disordered. Such states were calculated theoretically for viscous fingering back in the early 1990s by Ben Amar (albeit not their stability) and observed thereafter in experiments in sector geometries (Lajeunesse & Couder). For more details on this point, see the recent paper by Vaquero-Stainer et al Self-similar anddisordered front propagation in a radial Hele-Shaw channel with time-varying cell depth Phys. Rev. Fluids 4, 064002.

A. Juel

Reply: We thank the reviewer for bringing this up to our attention. In the revised paper, we revised the introduction and added the mentioned papers into the references.
